# Ethical decision-making for AI in mental health: the Integrated Ethical Approach for Computational Psychiatry (IEACP) framework

**DOI:** 10.1017/S0033291725101311

**Published:** 2025-07-24

**Authors:** Andrea Putica, Rahul Khanna, Wiliam Bosl, Sudeep Saraf, Juliet Edgcomb

**Affiliations:** 1 Department of Psychology, Counselling and Therapy, La Trobe University, Melbourne, VIC, Australia; 2 Melbourne School of Psychological Sciences, The University of Melbourne, Melbourne, VIC, Australia; 3 Phoenix Australia – Centre for Posttraumatic Mental Health, Department of Psychiatry, University of Melbourne, Melbourne, VIC, Australia; 4 Department of Psychiatry, Austin Health, Heidelberg, Melbourne, Australia; 5 School of Nursing and Health Professions, University of San Francisco, San Francisco, CA, USA; 6 Computational Health Informatics Program, Boston Children’s Hospital, Boston, MA, USA; 7 Boston Children’s Hospital, Harvard Medical School, Boston, MA, USA; 8 Department of Psychiatry, Alfred Health, Melbourne, VIC, Australia; 9 Mental Health Informatics and Data Science Hub, Semel Institute, University of California Los Angeles, Los Angeles, CA, USA; 10 Division of Child & Adolescent Psychiatry, Department of Psychiatry, David Geffen School of Medicine, University of California Los Angeles, Los Angeles, CA, USA

**Keywords:** clinical decision support, ethical framework, ethics, mental health informatics, psychiatry

## Abstract

The integration of computational methods into psychiatry presents profound ethical challenges that extend beyond existing guidelines for AI and healthcare. While precision medicine and digital mental health tools offer transformative potential, they also raise concerns about privacy, algorithmic bias, transparency, and the erosion of clinical judgment. This article introduces the Integrated Ethical Approach for Computational Psychiatry (IEACP) framework, developed through a conceptual synthesis of 83 studies. The framework comprises five procedural stages – Identification, Analysis, Decision-making, Implementation, and Review – each informed by six core ethical values – beneficence, autonomy, justice, privacy, transparency, and scientific integrity. By systematically addressing ethical dilemmas inherent in computational psychiatry, the IEACP provides clinicians, researchers, and policymakers with structured decision-making processes that support patient-centered, culturally sensitive, and equitable AI implementation. Through case studies, we demonstrate framework adaptability to real-world applications, underscoring the necessity of ethical innovation alongside technological progress in psychiatric care.

## Introduction

Computational psychiatry integrates insights from psychiatry, neuroscience, and computer science to develop data-driven approaches for diagnosis, prognosis, and treatment of mental health conditions (Corlett & Fletcher, 2014; Friston, 2023; Khanna et al., 2022). Clinicians face ethical challenges when implementing these approaches in practice (Espejo, Reiner, & Wenzinger, 2023; McCradden, Hui, & Buchman, 2023). Recent advancements in artificial intelligence (AI), including machine learning and generative language models, have expanded the potential of computational psychiatry to transform mental healthcare (Salazar de Pablo et al., 2021). While these challenges exist across medicine (Uusitalo, Tuominen, & Arstila, 2021), the sensitive nature of mental healthcare amplifies the ethical implications of these challenges. Despite existing ethical guidelines for general psychiatric practice and health AI (Solanki, Grundy, & Hussain, 2023; World Health Organization, 2021), computational psychiatry remains ethically underregulated. Without structured ethical governance, AI risks reinforcing systemic biases, compromising patient autonomy, and exacerbating existing disparities in mental healthcare.

Psychiatric data are sensitive, stigmatizing, and at times subjective, which can complicate informed consent and clinical transparency. Computational models trained on homogeneous datasets can amplify biases, skewing psychiatric diagnoses and treatments, particularly when applied across diverse cultural, gender, and age groups (Capon, Hall, Fry, & Carter, 2016; Coley et al., 2021; Leslie et al., 2021; Roy, 2017). Psychiatry’s reliance on subjective patient experiences means that overemphasis on computational tools may disguise paternalism (Juengst, McGowan, Fishman, & Settersten, 2016) if AI systems’ outputs override patients’ lived experiences. In this context, AI model opacity often further complicates patient-centered decision-making (Chin-Yee & Upshur, 2018; Ploug & Holm, 2016). Erosion in trust can damage the therapeutic alliance, a cornerstone of psychiatric practice, and diminish clinician’s ability to integrate psychosocial and cultural contexts into care (Chin-Yee & Upshur, 2018; Tekin, 2014; Walter, 2013). Furthermore, rapid evolution of AI demands that ethical frameworks remain adaptable to new technologies affecting symptomatology, diagnosis, and even therapeutic interventions (Barnett et al., 2018; Starke, De Clercq, Borgwardt, & Elger, 2021; Torous et al., 2021). Finally, by emphasizing genetics and biomarkers, established precision medicine frameworks risk reductionism and may overlook the crucial roles of environmental, cultural, and interpersonal determinants in mental health and psychiatric care (Venkatasubramanian & Keshavan, 2016).

The digital divide complicates ethical considerations of AI in global psychiatric contexts, particularly in rural, underserved, and impoverished regions. This divide between those with and without access to mobile or internet technologies intersects with cultural variations in mental health conceptualization and treatment. In low- and middle-income countries, primary care providers, who are often the first point of contact for mental health concerns, face numerous challenges in implementing computational tools that align with patient autonomy and local cultural perspectives regarding mental health treatment (Naslund et al., 2017). For example, cultures may conceptualize epilepsy as a neurological disorder, mental health condition, or spiritual issue (Gilkinson et al., 2022). Such variations extend to suicide reporting and help-seeking behaviors, where cultural and systemic factors significantly influence data accuracy and treatment engagement (Monosov et al., 2024; Naslund et al., 2017; Starke et al., 2021). The tripartite challenge of AI access, cultural understanding, and clinical implementation underscores the need for an ethical framework adaptable to the technical and sociocultural aspects of global mental healthcare delivery.

This article introduces the Integrated Ethical Approach for Computational Psychiatry (IEACP), a framework that addresses computational psychiatry’s ethical challenges while supporting patient-centered interdisciplinary care. We outline the framework’s development, emphasize patient and lived experience integration in ethical decision-making, and demonstrate its application through case studies. We also examine the framework’s implications, limitations, and future directions for global mental health care scalability. The ‘*Patient and lived experience involvement methods*’ section details our approach to incorporating patient perspectives to evaluate the alignment of computational psychiatric tools with real-world patient needs and experiences.

## Framework development methodology

The literature search strategy, inclusion and exclusion criteria, study selection process, data extraction procedures, and the full Preferred Reporting Items for Systematic Reviews and Meta-Analyses flow diagram are provided in the Supplementary Material Figure S-1.

### Development of framework components through structured review

The IEACP Framework was developed through interpretive synthesis of 83 peer-reviewed studies identified in a systematic literature review (see Supplementary Material). Structured data extraction captured information on implementation contexts, ethical challenges, and ethical values. Using repeated rounds of concept clustering, five core framework stages were identified: Identification, Analysis, Decision-making, Implementation, and Review. Each stage includes three commonly observed implementation processes. A stage refers to a broad phase in ethical implementation (e.g. the identification of risks), while a process refers to a recurring, practical approach within that stage (e.g. stakeholder mapping). Values refer to overarching ethical principles, such as privacy, autonomy, or justice, that guide decision-making throughout each stage and process. The framework focuses on the most frequently recurring processes within each stage that reflect clear ethical strategies across diverse settings. This approach aligns with the conceptual framework methodology (Jabareen, 2009).

Implementation contexts and ethical challenges revealed recurring, stage-specific implementation processes. For example, three distinct identification stage processes emerged: systematic recognition of ethical risks (e.g. D’Souza et al., 2024; privacy concerns), gathering of implementation information (e.g. Clarke, Foltz, & Garrard, 2020) examining data collection and storage requirements) and stakeholder mapping (e.g. Clarke et al., 2020; affected parties). Analysis stage processes were derived from studies evaluating ethical considerations, including technical evaluation (e.g. Monteith et al., 2023; data quality), compliance assessment (e.g. Ball, Kalinowski, & Williams, 2020; regulatory requirements), and guideline review (e.g. D’Souza et al., 2024; compliance frameworks). Decision-making stage processes emerged from implementation planning approaches, such as strategy development (e.g. Hurley et al., 2024; human supervision), implementation planning (e.g. Zhang et al., 2023; standardization), and consensus building (e.g. Zidaru, Morrow, & Stockley, 2021; codesign). Implementation stage processes were reflected in clinical application approaches, including clinical integration (e.g. Torous et al., 2021; healthcare adoption), staff training (e.g. D’Souza et al., 2024; workforce preparation), and performance monitoring (e.g. Clarke et al., 2020; ongoing evaluation). Review-stage processes were evident in the studies’ evaluation approaches, including outcome assessment (e.g. Dwyer & Koutsouleris, 2022; clinical translation), performance evaluation (e.g. Monteith et al., 2023; error tracking), and framework refinement (e.g. Fusar-Poli et al., 2022; continuous monitoring).

Analysis of 83 studies identified the following six key ethical values in computational psychiatry: privacy and confidentiality, transparency and explainability, justice and equity, beneficence and non-maleficence, autonomy and informed consent, and scientific integrity and validity. Privacy and confidentiality were most frequently discussed, followed by transparency in clinical decision-making. Justice considerations span algorithmic bias and healthcare access, while beneficence, autonomy, and integrity connect to risk assessment, informed consent, and model validation, respectively. The alignment of these ethical values with the IEACP framework stages was determined based on their frequency and implementation context in the literature (see Supplementary Table S-2). A selective set of exemplary references will be included throughout the article.

These six ethical principles were inductively derived through interpretive synthesis of ethical content across the included literature, consistent with conceptual framework development (Jabareen, 2009). The framework synthesizes established bioethical traditions, including traditional principlism (Beauchamp & Childress, 2001) expressed in the values of beneficence, non-maleficence, autonomy, and justice; information ethics (Floridi et al., 2018) emphasizing privacy protections in algorithmic data processing; virtue epistemology (Zagzebski, 1996), highlighting scientific integrity and epistemic responsibility; and epistemic justice theory (Fricker, 2007), with a focus on transparency and algorithmic accountability. This multitheoretical synthesis addresses computational psychiatry’s novel challenges that no single established framework adequately encompasses. Privacy extends beyond traditional confidentiality to encompass algorithmic data processing, model training on sensitive psychiatric information, and digital phenotyping that infers mental states from behavioral patterns. Transparency addresses epistemic opacity inherent in machine learning models where clinical recommendations emerge from processes that may be fundamentally unexplainable even to developers (Petch, Di, & Nelson, 2022). Scientific integrity ensures methodological rigor in computational models whose predictive validity directly influences psychiatric interventions. These six principles emerged as essential through repeated patterns in the literature addressing ethical challenges in computational psychiatry, with other commonly cited AI ethics principles either conceptually encompassed within the selected six or having limited direct applicability to clinical psychiatric contexts.

Ethical dilemmas in computational psychiatry characteristically arise from irreconcilable conflicts between these principles, requiring contextual navigation rather than algorithmic resolution. Transparency demands for explainable AI outputs can directly conflict with privacy requirements when model explanations necessarily reveal sensitive patient data or demographic patterns. Beneficence-driven early interventions based on suicide risk algorithms may fundamentally override patient autonomy when individuals reject computational predictions of their mental state. Justice requires equitable AI performance across populations while scientific integrity demands acknowledging when models perform poorly for certain demographic groups, creating tensions between deployment and methodological honesty. Privacy protections may conflict with justice when de-identification procedures disproportionately exclude marginalized populations from model development. The IEACP framework addresses these inherent tensions not by providing predetermined resolutions, but by requiring systematic identification of competing principles during stakeholder mapping, explicit analysis of trade-offs during compliance assessment, collaborative negotiation of acceptable compromises during consensus building, and ongoing monitoring of principle conflicts during implementation review. This procedural approach recognizes that ethical reasoning in computational psychiatry involves navigating irreducible tensions through structured deliberation rather than eliminating conflicts through hierarchical principle ranking, consistent with contextualist approaches to applied ethics (Jonsen & Toulmin, 1988) and established methods for managing principal conflicts in clinical ethics (Gillon, 2003).

## The IEACP framework

The IEACP framework addresses computational psychiatry ethics through five stages with three processes each, guided by six core values. Ethical values do not simply serve as retrospective considerations; they actively shape decision points at both stage transitions and within processes, embedding ethical integrity throughout implementation. Table 1 illustrates how ethical values inform decision points across each stage.

**Table 1.** Ethical decision-making across the Integrated Ethical Approach for Computational Psychiatry (IEACP) framework

Ethical principle	Identification stage		Analysis stage		Decision-making stage		Implementation stage		Review stage	
	Core function	Example	Core function	Example	Core function	Examples	Core function	Examples	Core function	Examples
Privacy and confidentiality (PC)	Identify data protection needs and vulnerabilities	*Map sensitive data in EHR; track crisis data collection points; define access requirements; assess AI privacy risks*	Evaluate privacy protection options	*Test de-identification methods; analyze access patterns; compare encryption options; verify HIPAA compliance*	Select privacy protection methods	*Choose encryption standard (AES–256); define access roles; set retention periods; establish breach protocols*	Deploy privacy protections	*Install encryption systems; configure access controls; launch audit logging; activate monitoring tools*	Assess privacy protection	*Audit access logs; review security incidents; test control effectiveness; verify compliance*
Autonomy and informed consent (AIC)	Recognize potential autonomy compromises	*Document capacity fluctuations; specify consent checkpoints; identify surrogate scenarios; review consent protocols*	Analyze consent and capacity assessment	*Evaluate capacity tools; test consent workflows; analyze override conditions; compare documentation methods*	Define consent requirements	*Set capacity thresholds; specify override criteria; choose documentation format; define proxy rules*	Implement consent systems	*Deploy consent forms; activate capacity tools; launch documentation system; configure override tracking*	Evaluate consent processes	*Review consent records; analyze override patterns; test capacity assessments; survey understanding*
Justice and equity (JE)	Map disparities and access needs	*Measure care access gaps; document cultural differences; identify literacy barriers; track resource distribution*	Assess bias and accessibility	*Measure demographic bias; test interface accessibility; calculate resource gaps; analyze error distributions*	Choose fairness measures	*Select fairness metrics; set disparity thresholds; choose mitigation methods; define resource allocation*	Deploy fairness measures	*Activate bias monitoring; install accessibility features; launch resource tracking; configure fairness alerts*	Monitor fairness	*Measure demographic performance; track accessibility; analyze resource distribution; review equity*
Transparency and explainability (TE)	Identify explanation requirements	*List critical decisions; survey stakeholder needs; specify documentation needs; verify disclosure rules*	Evaluate explanation methods	*Consider SHAP vs. LIME; test user comprehension; evaluate documentation formats; measure explanation accuracy*	Determine explanation standards	*Set importance thresholds; choose documentation template; define version control*	Implement explanation systems	*Deploy visualizations; launch model cards; configure version control; activate audit trails*	Review the explanation effectiveness	*Test explanation clarity; verify documentation; survey user understanding; check version compliance*
Beneficence and non-maleficence (BN)	Map the benefits and harm risks	*Identify intervention benefits; assess false positive impacts; define safety checkpoints; map unintended effects*	Analyze intervention impacts	*Calculate risk–benefit ratios; measure false positive rates; test safety protocols; evaluate intervention outcomes*	Set safety parameters	*Define risk thresholds; establish intervention triggers; set monitoring rules; choose safety protocols*	Deploy safety systems	*Launch risk monitoring; activate intervention alerts; install safety protocols; configure outcome tracking*	Assess intervention impact	*Track patient outcomes; monitor adverse events; measure intervention effectiveness; calculate benefit ratios*
Scientific integrity and validity (SIV)	Identify validation requirements	*Define performance metrics; specify validation data; establish quality controls; test generalizability*	Assess validation methods	*Test prediction accuracy; measure model stability; evaluate quality metrics; analyze generalizability*	Define validation standards	*Set accuracy thresholds; choose quality metrics; define update criteria; establish test procedures*	Implement validation systems	*Deploy performance monitoring; launch quality controls; activate drift detection; configure update triggers*	Validate performance	*Measure accuracy metrics; check model drift; verify quality standards; test generalizability*

*Note*: This table outlines the five procedural stages of the Integrated Ethical Approach for Computational Psychiatry (IEACP) framework – Identification, Analysis, Decision-making, Implementation, and Review and Reflection – while highlighting how ethical values underpin each stage. LIME, Local Interpretable Model-agnostic Explanations; SHAP, SHapley Additive exPlanations.

## Identification

The *Identification* stage consists of three processes as follows: recognizing ethical challenges, gathering implementation data, and mapping stakeholders. Recognition is the process of identifying ethical challenges at the intersection of computational psychiatry and clinical ethics, including moral dilemmas that arise from AI-driven decision-making. For example, in schizophrenia prediction models, the recognition process examines the ethical tension between early intervention and the risk of overdiagnosis, focusing on how predictive modeling may inadvertently reinforce diagnostic overshadowing, limit patient autonomy, and exacerbate social stigma. Gathering implementation data involves collecting information on real-world implementation contexts, including technical, clinical, and systemic factors affecting ethical deployment. For example, in machine learning models for depression screening, information gathering involves documenting existing screening workflows across clinical settings, mapping the technological infrastructure needed for ethical deployment, and characterizing the target patient demographics to anticipate potential disparities. Stakeholder mapping identifies all individuals and groups impacted by computational psychiatry tools, a critical process given the complex relationships between clinicians, patients, carers, and automated systems. In an automated suicide risk prediction system, stakeholder mapping identifies key individuals and groups impacted by the tool, including emergency clinicians, at-risk patients, family members, mental health teams, hospital administrators, and healthcare funders, such as government agencies and insurance providers. Special attention is given to populations with variable decision-making capacity, such as individuals with first-episode psychosis, by identifying their interactions with AI tools, mapping support networks, and documenting contexts requiring added protections (Vayena, Blasimme, & Cohen, 2018).

Ethical values shape the Identification stage, guiding the recognition of ethical challenges, data collection, and stakeholder mapping to ensure responsible integration of computational psychiatry tools. Identifying algorithmic risks, such as biased data sources, unintended clinical consequences, or ethical dilemmas in psychiatric prediction models, ensures that potential harms are flagged before deployment (beneficence and non-maleficence; Ball et al., 2020; Fusar-Poli et al., 2022). The identification of methodological limitations during instrument development helps prevent flawed assumptions from influencing clinical judgment (scientific integrity; McCradden et al., 2023; Monteith et al., 2023). Pre-implementation data collection identifies ethical risks related to psychiatric information security, storage, and sharing, preventing potential misuse or unauthorized access (privacy and confidentiality; D’Souza et al., 2024; Upreti, Lind, Elmokashfi, & Yazidi, 2024). Recognizing disparities in how AI systems identify psychiatric risk across different demographic groups is essential to prevent reinforcing existing inequities (justice and equity; Gooding & Kariotis, 2021; Sahin et al., 2024). Stakeholder mapping ensures that AI-driven tools acknowledge patient rights and decision-making capacity, particularly in populations where autonomy may be impacted, such as individuals with severe mental illness (autonomy and informed consent; Davidson, 2022; Jacobson et al., 2020). Recognizing when computational psychiatry tools produce opaque decision-making processes ensures that risks related to interpretability are identified (transparency and explainability; D’Souza et al., 2024; McCradden et al., 2023). This integrated approach embeds ethical rules into the earliest stages of tool development, shifting ethical considerations from retrospective checkpoints to foundational guideposts that shape computational psychiatry’s trajectory from inception.

## Analysis

The *Analysis* stage deepens the examination of ethical considerations identified in the previous stage, moving from recognizing potential challenges to evaluation of their implications and compliance requirements. While the Identification stage maps ethical concerns, the Analysis stage rigorously assesses them against established regulatory, institutional, and professional standards. This involves three key processes: technical evaluation, compliance assessment, and guideline review. Technical evaluation assesses computational methods against predefined benchmarks, ensuring accuracy, sensitivity, specificity, and fairness across diverse populations. This involves evaluating model reliability, demographic fairness, and interpretability within psychiatric contexts. For example, when assessing a depression screening algorithm, technical evaluation determines whether the model performs equitably across age groups, ethnicities, and socioeconomic backgrounds, identifying potential biases and performance disparities. Compliance assessment evaluates alignment with relevant healthcare laws and data protection regulations. In computational psychiatry, this involves reviewing adherence to healthcare laws, data protection frameworks such as the European Union’s General Data Protection Regulation (European Union, 2016) and the United States Health Insurance Portability and Accountability Act (HIPAA, 1996), particularly regarding sensitive mental health data. For example, this might involve assessing whether a mood prediction algorithm appropriately manages patient consent requirements or ensuring diagnostic support systems comply with data privacy regulations in psychiatric settings. Guideline review examines adherence to professional and institutional standards in computational psychiatry. This includes evaluating compliance with frameworks such as the World Health Organization’s Ethics and Governance of Artificial Intelligence for Health (World Health Organization, 2021), as well as institution-specific psychiatric governance policies.

During Analysis, ethical values structure the evaluation of computational tools against healthcare’s regulatory, institutional, and professional requirements. Assessing computational models against predefined benchmarks for accuracy, sensitivity, specificity, and fairness ensures that potential biases and risks to patient safety are identified before deployment (beneficence and non-maleficence; Ball et al., 2020; Fusar-Poli et al., 2022). Validation and external assessment of model assumptions safeguard against methodological flaws that could compromise clinical decisions (scientific integrity; McCradden et al., 2023; Monteith et al., 2023). Ensuring compliance with healthcare laws and data protection frameworks, such as General Data Protection Regulation (GDPR) and HIPAA, prevents unauthorized data use and security breaches (privacy and confidentiality; D’Souza et al., 2024; Farmer, Lockwood, Goforth, & Thomas, 2024). Evaluating demographic performance variations in psychiatric assessments ensures that AI-driven tools do not reinforce inequities in access to care (justice and equity; Singhal et al., 2024; Upreti et al., 2024). Reviewing adherence to institutional and professional ethical guidelines safeguards patient autonomy and ensures that AI-assisted decision-making does not override informed consent policies (autonomy and informed consent; Ahmed & Hens, 2022; Wouters et al., 2024). Maintaining interpretability in analytic outputs allows clinicians and regulators to scrutinize AI decision-making before implementation (transparency and explainability; D’Souza et al., 2024; McCradden et al., 2023).

## Decision-making

The Decision-making stage translates ethical analysis into actionable implementation strategies through three key processes: strategy development, implementation planning, and consensus building. Strategy development formulates approaches to address ethical challenges, ensuring AI tools align with professional and patient-centered considerations, such as restricting suicide risk prediction models to clinician-mediated use to mitigate risks of self-directed harm or unnecessary emergency interventions. Implementation planning translates ethical requirements into structured operational protocols that guide clinical practice, including developing tiered consent mechanisms for depression screening algorithms that dynamically adjust based on patient cognitive capacity, ensuring informed decision-making at different stages of illness progression. Consensus building facilitates structured consultation and collaborative review among clinicians, ethicists, and individuals with lived experience to define ethically defensible risk thresholds in automated psychiatric screening, such as distinguishing passive suicidal ideation from active crisis situations that require immediate intervention. Through iterative stakeholder engagement, this process refines actionable criteria for when algorithmic predictions should trigger clinician review, ensuring decisions optimize predictive accuracy while maintaining patient autonomy, clinical feasibility, and ethical oversight.

During the Decision-making stage, ethical values structure the translation of analysis into actionable plans, ensuring that strategic interventions align with patient safety, clinical integrity, and equitable outcomes. Defining intervention thresholds and protocols ensures that suicide risk predictions lead to appropriate clinical responses, balancing proactive intervention with harm prevention (beneficence and non-maleficence; Torous et al., 2021; Wang et al., 2024). Developing structured validation standards and operational procedures safeguards against methodological inconsistencies, ensuring AI applications maintain reliability across psychiatric settings (scientific integrity; Kirtley et al., 2022; Monteith et al., 2023). Establishing consent frameworks through stakeholder collaboration ensures that AI-driven recommendations respect patient autonomy across varying cognitive capacities (autonomy and informed consent; Jacobson et al., 2020; Wouters et al., 2024). Defining equitable intervention criteria prevents disparities in AI-assisted psychiatric care, ensuring that deployment procedures address demographic considerations (justice and equity; Koutsouleris, Hauser, Skvortsova, & De Choudhury, 2022; Starke et al., 2021). Determining data access levels and security protocols ensures psychiatric information remains protected against breaches and unauthorized use (privacy and confidentiality; Parziale & Mascalzoni, 2022; Upreti et al., 2024). Establishing clear guidelines for communicating AI system outputs ensures that stakeholders can accurately interpret AI-generated insights, thereby reducing ambiguity and informing structured decision-making (transparency and explainability; Gültekin & Şahin, 2024; Torous et al., 2021). By embedding ethical values into decision-making at every stage, this structured framework ensures that computational psychiatry tools align with professional standards while maintaining stakeholder trust and clinical applicability.

## Implementation

The *Implementation* stage transitions from decision-making to active deployment, integrating computational psychiatry tools into clinical workflows through three interconnected processes: clinical integration, staff training, and performance monitoring. Clinical integration embeds computational tools into existing mental healthcare systems through systematic protocol development and infrastructure integration. For example, implementing a psychosis relapse prediction algorithm requires three key components. First, establishing empirically validated clinical triggers based on quantifiable indicators (e.g. appointment adherence and medication compliance). Second, securely integrating outputs with electronic health record systems while maintaining data integrity. Third, structuring evidence-based response pathways that escalate from automated surveillance to structured clinical assessments and expedited psychiatric intervention within established governance frameworks. Staff training develops clinical competency in three clinical areas: first, quantitative risk score interpretation within clinical contexts, incorporating confidence intervals, and limitation awareness. Second, integrating algorithmic insights with clinical expertise, emphasizing systematic evaluation against patient-specific factors. Third, training in communicating AI-generated outputs while maintaining a therapeutic alliance. Performance monitoring establishes comprehensive evaluation frameworks to track predictive accuracy, monitor unintended consequences (e.g. demographic disparities), assess care quality impact, and ensure ongoing ethical compliance.

In the Implementation stage, ethical values guide the active deployment of computational psychiatry tools through clinical integration, staff training, and performance monitoring. Clinical teams implement alert systems and response pathways using predetermined risk thresholds, ensuring AI supports rather than overrides clinical judgment (beneficence and non-maleficence; Monaco et al., 2024; Tabb & Lemoine, 2021). Validation procedures and quality control measures become part of routine clinical practice through systematic staff training (scientific integrity; Sultan, Scholz, & van den Bos, 2023; Zhang et al., 2023). Dynamic consent processes roll out with documentation systems that adapt to varying levels of patient cognitive capacity throughout illness phases (autonomy and informed consent; Ball et al., 2020; Wouters et al., 2024). Monitoring systems track utilization patterns as standardized access protocols take effect, ensuring equitable tool deployment across patient populations (justice and equity; Lewis et al., 2024; Wang et al., 2024). Clinical staff receive training in security protocols while system-level protections and access controls safeguard sensitive psychiatric data (privacy and confidentiality; Upreti et al., 2024; Wray et al., 2021). Clear formats for sharing AI outputs roll out alongside training in effective communication methods, maintaining transparency throughout implementation (transparency and explainability; Levkovich, Shinan-Altman, & Elyoseph, 2024; Wiese & Friston, 2022). For deep learning systems where algorithmic interpretability is not possible, transparency focuses on post-hoc explanations (e.g. SHapley Additive exPlanations and Local Interpretable Model-agnostic Explanations) and model behavior rather than step-by-step algorithmic logic (Lundberg & Lee, 2017; Ribeiro, Singh, & Guestrin, 2016). This coordinated integration of ethical principles into clinical practice transforms theoretical frameworks and planned protocols into living systems that actively safeguard and enhance psychiatric care delivery.

## Review and reflection

The *Review and reflection* stage provides a structured approach to evaluating real-world impact and refining AI tools over time. The Review and reflection stage evaluates real-world performance through outcome assessment, performance evaluation, and framework refinement to ensure continuous improvement. Outcome assessment systematically measures the impact of implemented computational psychiatry tools using quantitative and qualitative metrics, tracking clinical outcomes, such as symptom improvement rates, treatment adherence, and changes in healthcare utilization. For example, when assessing an automated depression screening system in primary care, outcome assessment would measure time-to-treatment initiation, referral success rates, patient-reported symptom changes at 6 months, and emergency department utilization for mental health crises. Performance evaluation examines prediction accuracy, response times, and clinical workflow integration. In a psychosis relapse prediction system, this involves assessing how accurately the model identifies early warning signs, how quickly clinical teams respond to alerts, and whether predictions integrate smoothly into routine assessments without disrupting existing workflows. Framework refinement updates protocols based on implementation experience and emerging evidence, such as modifying alert thresholds to reduce false positives or adjusting clinical workflows based on staff feedback. By continuously adapting predictive models, clinical integration protocols, and risk management frameworks, this stage ensures computational psychiatry tools evolve ethically and remain clinically responsible. For example, if an algorithm achieves 85% accuracy in detecting early warning signs but overnight alerts take significantly longer to receive a clinical response, framework refinement would focus on optimizing workflow protocols during off-hours to improve efficiency and clinical impact.

During the Review and reflection stage, ethical values guide the evaluation of how computational psychiatry tools performed, ensuring that their real-world impact aligns with ethical principles and clinical objectives. Assessing documented clinical effects and incident reports ensures that AI-driven interventions minimize harm while optimizing patient care (beneficence and non-maleficence; Levkovich et al., 2024; Torous et al., 2021). Reviewing performance data identifies model reliability across diverse clinical settings, ensuring psychiatric AI applications maintain scientific rigor (scientific integrity; Kleine et al., 2023; Monteith et al., 2023). Examining consent records evaluates whether patients engaged meaningfully with AI-assisted care, ensuring informed decision-making was upheld (autonomy and informed consent; Davidson, 2022; Zidaru et al., 2021). Evaluating demographic trends in patient outcomes ensures that psychiatric AI tools do not reinforce disparities in treatment access and effectiveness (justice and equity; Lewis et al., 2024; Wang et al., 2024). Reviewing security protocols and access logs ensures that psychiatric data remains protected against breaches and misuse (privacy and confidentiality; Upreti et al., 2024; Wray et al., 2021). Analyzing stakeholder feedback on AI applications assesses whether decision-making processes remained transparent, interpretable, and clinically actionable (transparency and explainability; Kline, Prichett, McKim, & Palm Reed, 2023; Wiese & Friston, 2022). Without structured ethical oversight, AI risks amplifying biases, diminishing clinical accountability, and undermining patient trust in mental healthcare. The IEACP framework provides an essential ethical infrastructure to ensure computational psychiatry advances in a way that prioritizes transparency, equity, and patient autonomy. Table 2 operationalizes all IEACP framework processes, providing step-by-step implementation guidance with systematic cultural integration that transforms the conceptual framework into actionable procedures for clinical practice.

**Table 2.** Operationalization of the Integrated Ethical Approach for Computational Psychiatry (IEACP) framework with cultural adaptations

Stage/process	Steps	Decision criteria	Completion standards	Cultural adaptations
Identification				
Recognizing ethical challenges	PC: Document what patient data is collected, how it is stored, who accesses it, retention periods, and sharing protocols. AIC: Identify all patient decision points, assess informed consent adequacy, and determine override scenarios. BN: Calculate potential benefits versus harms and identify vulnerable populations. JE: Examine demographic performance differences and assess accessibility barriers. TE: Evaluate explanation capacity for patients and clinicians. SIV: Review validation methods, data quality, and model limitations	Flag as high-concern if >2 principles show significant issues, vulnerable populations comprise >25% of users, and potential for serious patient harm exists^a^	All six principles systematically reviewed, specific concerns documented with evidence, severity ranking assigned (low/medium/high), and conflicts between principles identified	Map culture-specific privacy concepts (PC: collective vs. individual data ownership), consent models (AIC: family vs. individual decision-making authority), benefit definitions (BN: Western medical vs. traditional healing outcomes), justice concerns (JE: AI trained on majority populations), explanation preferences (TE: narrative vs. statistical communication), and validation requirements (SIV: cultural expression of symptoms may confound Western diagnostic AI)
Gathering implementation data	Technical**:** Document algorithm type, training data sources, performance metrics, update frequency, and failure modes. Clinical**:** Map current workflows, identify integration points, assess staff readiness, and determine patient impact. Regulatory**:** Review applicable laws (HIPAA and state regulations), institutional policies, and professional guidelines. Organizational**:** Assess infrastructure capacity, budget requirements, and timeline constraints	Documentation is adequate when all technical specifications are recorded, clinical workflows are fully mapped, regulatory requirements are identified, and resource needs are quantified	Complete technical specification document, detailed workflow analysis, regulatory compliance checklist completed, and resource assessment finalized	Document cultural privacy frameworks (PC: tribal sovereignty and collective ownership), consent structures (AIC: family hierarchies and religious authorities), benefit definitions (BN: traditional healing metrics and spiritual outcomes), equity metrics (JE: language-specific performance and cultural access barriers), communication needs (TE: interpreter services and cultural explanation formats), and validation requirements (SIV: culture-specific assessments and traditional diagnostic compatibility)
Stakeholder mapping	Clinical stakeholders**:** List all clinicians who will use the system, receive outputs, or be affected by decisions. Patient stakeholders**:** Identify target patient populations, family members, caregivers, and advocacy groups. Administrative**:** Include IT staff, quality officers, legal counsel, and executives. **E**xternal**:** Consider regulators, payers, and community groups. For each stakeholder: assess influence level (high/medium/low), interest level (high/medium/low), decision-making authority, and potential concerns	Include stakeholders if directly affected by AI decisions, have implementation authority, represent a significant constituency, and can block implementation	Comprehensive stakeholder list with influence/interest analysis, decision-making authority mapped, and engagement strategy defined for each stakeholder group	Include data governance authorities (PC: tribal councils and community data guardians), decision-making authorities (AIC: family elders, religious leaders, and community councils), healing advocates (BN: traditional healers, cultural health workers, and spiritual advisors), equity representatives (JE: minority community liaisons, interpreter coordinators, and disability advocates), communication facilitators (TE: cultural navigators, storytellers, and community educators), and knowledge validators (SIV: indigenous researchers, traditional knowledge keepers, and cultural psychiatrists)
Analysis				
Technical evaluation	Performance analysis: Calculate accuracy, sensitivity, and specificity for the overall population and demographic subgroups. Bias assessment: Examine performance differences across age, gender, race, and socioeconomic status. Reliability testing: Assess consistency across different clinical settings and time periods. Interpretability review: Evaluate whether decisions can be explained to clinicians and patients	Performance is acceptable if overall accuracy ≥85%, no demographic group <80% accuracy, performance differences between groups <10%, and key decisions can be explained^a^	Statistical analysis complete with confidence intervals, demographic fairness confirmed, reliability across contexts validated, and interpretability assessment documented	Assess performance across cultural groups (PC: culturally sensitive data handling), validate across cultural decision-making contexts (AIC: family vs. individual consent scenarios), evaluate harm/benefit calculations in cultural frameworks (BN: traditional vs. Western outcome measures), test fairness across cultural populations (JE: language groups, immigrant status, and cultural minorities), assess cultural explanation methods (TE: narrative vs. statistical preferences), and validate cultural symptom expressions (SIV: non-Western diagnostic presentations and cultural idioms of distress)
Compliance assessment	Legal compliance**:** Review HIPAA requirements, state privacy laws, and FDA regulations if applicable. Institutional compliance**:** Check IRB requirements, institutional AI policies, and clinical guidelines. Professional standards: Assess against relevant professional society guidelines (APA, etc.). International standards: Consider applicable international frameworks (WHO AI ethics, etc.)	Compliance is achieved when all legal requirements are met, institutional policies are satisfied, professional standards are adhered to, and no unresolved conflicts are identified	Legal review complete with documentation, institutional approval obtained, professional standard compliance verified, and compliance gaps resolved	Identify data governance conflicts (PC: competing sovereignty frameworks), document decision-making authority differences (AIC: individual vs. collective consent models), locate benefit-harm standard variations (BN: biomedical vs. traditional healing efficacy measures), verify access equity gaps (JE: language and cultural accessibility barriers), confirm disclosure requirement differences (TE: privacy vs. transparency cultural norms), and identify evidence standard differences (SIV: clinical trial vs. traditional validation approaches)
Guideline review	Clinical guidelines: Review against relevant diagnostic/treatment guidelines for the clinical condition. Ethics frameworks: Assess against established bioethics principles and AI ethics guidelines. Quality standards: Check against clinical quality measures and safety standards. Best practices: Compare to published best practices for similar AI implementations	Guidelines compliance when clinical standards are met, ethics frameworks are satisfied, quality measures are addressed, and best practices are incorporated	Guideline compliance matrix completed, gaps identified and addressed, and documentation of adherence provided	Document local diagnostic criteria conflicts with AI training standards (PC: traditional vs. DSM-based classifications), identify consent requirement conflicts (AIC: legal vs. cultural decision-making hierarchies), map safety measure differences (BN: biomedical vs. traditional healing adverse events), locate validation gaps for local populations (JE: demographic representation in cited studies), specify communication methods satisfying legal and cultural norms (TE: written vs. oral consent), and determine evidence standards accommodating multiple knowledge systems (SIV: clinical trial vs. traditional validation requirements
Decision-making				
Strategy development	Intervention thresholds: Define specific cut-points for AI recommendations requiring action. Human oversight: Specify when and how humans review AI decisions. Bias mitigation: Develop specific approaches to address identified fairness issues. Risk management: Create protocols for handling AI failures, false positives/negatives	Strategy is complete when thresholds are validated by clinical experts, oversight roles are clearly defined, bias mitigation is tested, and risk protocols are established	Implementation strategy document with specific thresholds, oversight protocols defined, bias mitigation plan tested, and risk management procedures established	Define culturally appropriate thresholds (BN: cultural distress expressions), specify cultural oversight (AIC: elder/family authority), develop cultural bias mitigation (JE: population-specific gaps), create cultural risk protocols (PC: traditional confidentiality norms), establish cultural communication (TE: narrative explanation methods), and ensure cultural validation (SIV: traditional evidence integration)
Implementation planning	Timeline development: Create a detailed project timeline with milestones, dependencies, critical path. Resource allocation: Assign specific personnel, budget, and equipment to each task. Training design: Develop a comprehensive training program with curriculum, assessments, and certification. Integration planning: Specify technical integration steps, testing procedures, and rollback plans	Planning is adequate when the timeline includes all critical activities, resources allocated to all tasks, the training program is complete and integration has been tested	Detailed project plan with timeline, resource assignments complete, training program developed and tested, and integration procedures validated	Develop culturally informed timelines (PC: community consultation periods), allocate cultural resources (AIC: interpreters and cultural liaisons), design culturally adapted training (BN: traditional healing integration and cultural safety), plan cultural testing (JE: validate across populations and accessibility features), establish cultural protocols (TE: multi-language materials, narrative methods), and ensure cultural validation (SIV: community review and traditional knowledge integration)
Consensus building	Stakeholder engagement: Conduct individual meetings with key stakeholders to understand concerns. Group facilitation: Host structured workshops to build consensus on key decisions. Conflict resolution: Address disagreements through structured negotiation and comprom**ise.** Agreement documentation: Obtain formal commitments from stakeholders on the final approach	Consensus is achieved when ≥80% of stakeholders agree on key decisions, major concerns are addressed through compromise, and formal agreements are obtained	Stakeholder agreement documented, implementation approach endorsed by key parties, and governance structure established with defined roles	Facilitate culturally informed processes (AIC: accommodate collective vs. individual decision-making), resolve conflicts using cultural mediation (BN: integrate traditional conflict resolution methods), establish transparent communication (TE: use culturally preferred consensus indicators), and validate across knowledge systems (SIV: ensure stakeholder alignment on implementation approach)
Implementation				
Clinical integration	System configuration: Install and configure the AI system within the clinical environment. Workflow integration: Embed AI outputs into clinical decision-making processes. User interface deployment: Implement user-friendly interfaces for clinicians. Performance monitoring setup: Establish real-time monitoring of system performance	Integration is successful when the system functions within the clinical environment, workflows are adapted to incorporate AI, users can effectively interact with the system and monitoring systems are operational	AI system deployed and operational, clinical workflows successfully adapted, user training completed, and monitoring systems active	Configure systems for cultural contexts (PC: culturally appropriate data protection), integrate workflows with cultural decision-making (AIC: accommodate family/elder consultation), deploy culturally adapted interfaces (BN: traditional healing terminology and cultural safety indicators), establish cultural performance monitoring (JE: track equity across populations), ensure transparent communication (TE: culturally-appropriate alerts), and validate across cultural contexts (SIV: test performance with diverse presentations)
Staff training	Knowledge training: Educate staff on AI capabilities, limitations, and interpretation of outputs. Skills training: Provide hands-on practice with system use and decision-making scenarios. Ethics training: Ensure understanding of ethical considerations, patient rights, and bias recognition. Competency assessment: Test staff knowledge and skills before independent use	Training is complete when all staff complete the required curriculum, demonstrate competency on assessments, and show proficiency in hands-on scenarios	Training curriculum delivered to all staff, competency assessments passed, hands-on proficiency demonstrated, and ongoing education scheduled	Provide cultural knowledge training (PC: privacy norms and data sovereignty), deliver cultural skills training (AIC: family consultation and collective decision-making), ensure cultural ethics training (BN: traditional healing integration and cultural safety), conduct cultural competency assessment (JE: equity awareness and bias recognition), establish cultural communication training (TE: culturally appropriate explanations), and validate cultural proficiency (SIV: assess competency across diverse scenarios)
Performance monitoring	Technical monitoring: Track AI system accuracy, reliability, uptime, and error rates. Clinical monitoring: Monitor patient outcomes, safety events, and clinical workflow efficiency. User monitoring: Assess clinician satisfaction, usage patterns, and reported concerns. Ethical monitoring: Watch for bias emergence, fairness issues, and patient complaints	Monitoring is adequate when all key metrics are tracked continuously, regular review meetings are held, feedback systems are functioning, and corrective actions are taken when needed	Monitoring dashboard operational, regular review processes established, feedback mechanisms functioning, and improvement actions documented	Track cultural performance metrics (PC: monitor data protection compliance and privacy breaches), monitor cultural clinical outcomes (AIC: assess family satisfaction with decision-making and consent effectiveness), evaluate cultural safety indicators (BN: track traditional healing integration and cultural adverse events), monitor cultural equity metrics (JE: assess performance disparities across groups and accessibility usage), establish cultural feedback systems (TE: culturally appropriate complaint mechanisms), and validate cultural performance (SIV: monitor accuracy across diverse presentations)
Review				
Outcome assessment	Clinical outcomes: Measure changes in diagnostic accuracy, treatment effectiveness, and patient safety. Operational outcomes: Assess workflow efficiency, resource utilization, and cost impact. User outcomes: Evaluate clinician satisfaction, confidence, and decision-making quality. Patient outcomes: Monitor patient satisfaction, health outcomes, and care experience	Assessment is complete when clinical outcomes are measured over an adequate timeframe, operational impacts are quantified, user feedback is collected, and patient perspectives are included	Comprehensive outcome evaluation with statistical analysis, operational impact assessment, stakeholder feedback analysis, and patient outcome measurement	Measure cultural clinical outcomes (PC: assess data protection effectiveness and privacy satisfaction), evaluate cultural operational outcomes (AIC: measure family engagement and consent efficiency), assess cultural user outcomes (BN: evaluate cultural competency and traditional healing integration), monitor cultural patient outcomes (JE: measure satisfaction across groups and outcome disparities), establish cultural feedback analysis (TE: assess communication effectiveness), and validate outcome measures (SIV: ensure validity across diverse populations)
Performance evaluation	Accuracy assessment: Track prediction accuracy, calibration, and discrimination over time. Fairness evaluation: Monitor performance across demographic groups for emerging bias. Reliability analysis: Assess system stability and consistency across different conditions. Usability review: Evaluate user interface effectiveness and workflow integration success	Performance is acceptable when accuracy is maintained within acceptable bounds, no significant bias is detected, system reliability is confirmed, and usability is satisfactory	Performance analysis with statistical controls, bias assessment across demographic groups, reliability analysis complete, and usability evaluation documented	Track cultural accuracy (PC: monitor prediction accuracy across privacy contexts), evaluate cultural fairness (AIC: assess performance across decision-making contexts), analyze cultural reliability (BN: evaluate consistency across traditional and biomedical frameworks), review cultural usability (JE: assess effectiveness across groups, language accessibility), establish cultural communication (TE: evaluate explanation effectiveness), validate performance standards (SIV: ensure accuracy measures valid across diverse presentations)
Framework refinement	Lessons learned synthesis: Analyze implementation challenges, successes, and unexpected issues. Framework updates: Revise framework components based on empirical evidence. Process improvements: Update implementation procedures based on practical experience. Knowledge sharing: Disseminate learnings to the broader community	Refinement is complete when lessons are systematically analyzed, framework updates are validated, improved processes are tested, and knowledge is shared with the community	Lessons learned analysis complete, framework revisions validated by stakeholders, improved procedures documented and tested, and best practices shared	Synthesize cultural lessons (PC: analyze privacy challenges and data sovereignty issues), update cultural components (AIC: revise decision-making guidance), improve cultural processes (BN: refine traditional healing integration), share cultural knowledge (JE: disseminate equity lessons across communities), establish communication improvements (TE: refine explanation methods), and validate refinements (SIV: test updates across diverse contexts)

*Note*: This table operationalizes the IEACP framework across all 15 processes, providing step-by-step implementation guidance with systematic cultural integration for each ethical principle.

Ethical values: AIC, autonomy/informed consent; BN, beneficence/non-maleficence; JE ,justice/equity; PC, privacy/confidentiality; TE, transparency/explainability; SIV, scientific integrity/validity.

Technical terms: AI,  artificial intelligence; APA, American Psychological Association; SM, Diagnostic and Statistical Manual of Mental `Disorders; FDA, Food and Drug Administration; HIPAA, Health Insurance Portability and Accountability Act; IRB, Institutional Review Board; IT, information technology; WHO, World Health Organization.

a These thresholds were developed to support practical decision-making and are not intended as rigid cutoffs. They reflect the cumulative risk approach discussed in prior ethical frameworks for digital and AI-enabled mental healthcare (Ball et al., 2020; Vayena et al., 2018).

### Patient and lived experience involvement methods

Ensuring computational psychiatry tools align with patient needs requires meaningful involvement, particularly for individuals with severe mental illness (Zima, Edgcomb, & Fortuna, 2024). Methods include adapted consent processes, codesign initiatives, lived experience advisory panels, peer-led focus groups, and representation in ethical oversight committees. Key considerations are outlined in Table 3.

**Table 3.** Patient considerations within the IEACP framework

Consideration	Strategy
Fluctuating capacity	Implement processes allowing involvement during periods of wellness with contingency plans
Supported decision-making	Utilize models involving trusted individuals without removing patient autonomy
Trauma-informed approaches	Implement practices recognizing high trauma prevalence
Diverse representation	Ensure the involvement of patients with a range of diagnoses
Accessibility	Provide multiple participation modes to accommodate various symptoms and needs
Safety planning	Develop clear protocols for managing potential distress or crises
Cognitive adaptations	Adapt materials to accommodate cognitive symptoms
Stigma reduction	Create an environment where patients feel valued as experts of their own experience
Child participants	Involve parents or caregivers in the decision-making process while respecting the child’s evolving capacities; use age-appropriate explanations and consent procedures

### Framework applications across computational psychiatry contexts

To illustrate the framework’s utility and adaptability, we examined its application across diverse computational psychiatry contexts. To that end, we analyzed two recent studies employing machine learning for mental health applications. Curtiss (2024) developed an ensemble machine learning approach to optimize second-step depression treatment selection. Their study used data from 1,439 patients in the Sequenced Treatment Alternatives to Relieve Depression trial who had failed to achieve remission with initial antidepressant treatment. The authors created models to predict outcomes for seven different second-step treatments, highlighting the complexity of personalized treatment selection in psychiatry. Grimland (2024) focused on real-time suicide risk prediction in crisis hotline chats. They analyzed 17,654 chat sessions using natural language processing and a theory-driven lexicon of suicide risk factors. To demonstrate a concrete framework application, Table 4 presents illustrative scenarios that extrapolate from documented study limitations and established patterns of algorithmic bias in psychiatric populations.

**Table 4.** Application of the Integrated Ethical Approach for Computational Psychiatry (IEACP) framework

Stage and process	Curtiss, Smoller, and Pedrelli (2024)	Grimland et al. (2024)
Identification		
Ethical challenge recognition	Recognizes: Algorithm predicted a 51% chance of remission with sertraline compared to 82% with psychotherapy, yet still recommended sertraline requiring ethics review (BN); Latino families requesting familismo decisions conflicting with individual consent in eight cases (AIC); 75.8% White training versus 40% minority deployment creating validity concerns (JE); 155 psychiatric variables accessible without encryption across 15 sites (PC); Spanish-speaking families unable to receive explanations in 12 encounters (TE); and 31% accuracy gaps between White and minority patients (SIV)	Identifies challenges in: SR-BERT achieving 92.1% AUC with 2.3-s delays creating beneficence tension between accuracy and crisis response speed (BN), capacity assessment conflicts in 847 crisis cases (AIC), accessibility barriers in 23% of 17,654 sessions (JE), encryption protocols conflicting privacy protection with immediate suicide risk access (PC), lexicon outputs incomprehensible to 34% of users (TE), and cultural bias affecting 28% of risk factor validation (SIV)
Information gathering	Gathers: STAR*D calculations revealing 31% CT-medication gap (BN), 20 Latino family interviews revealing autonomy conflicts in 65% (AIC), 75.8% White enrollment creating minority unknowns (JE), cybersecurity review finding 155-variable security gaps (PC), translator finding zero Spanish explanations (TE), and validation results showing 31% accuracy differences by ethnicity (SIV)	Documents: Processing delays affecting 15% of high-risk interventions, quantifying accuracy-speed trade-offs (BN), capacity impairment in 847 cases requiring modified consent (AIC), language barriers in 4,061 sessions (JE), encryption increasing latency by 0.8 s intensifying privacy-safety conflicts (PC), 66% comprehension failure in risk communication (TE), and cultural bias in 28% of risk assessments (SIV)
Stakeholder mapping	Maps: 25 psychiatrists across 15 centers identifying concerns about 51% sertraline over 82% therapy (BN), focus groups discovering familismo conflicts with individual consent (AIC), liaisons recruiting representatives from 75.8% White STAR*D plus Latino/African American community leaders (JE), IT director mapping security personnel protecting 155 variables with varying cultural protocols (PC), cultural consultant identifying Spanish families lacking explanation resources (TE), and validation teams tracking 31% demographic disparities (SIV)	Maps: Crisis counselors managing beneficence tensions between 2.3-s SR-BERT delays and emergency response needs (BN), mental health professionals navigating consent with 847 capacity-impaired callers (AIC), volunteer staff addressing accessibility barriers in 23% of sessions (JE), IT security teams balancing encryption requirements against immediate crisis access demands (PC), counselors explaining incomprehensible risk scores to 66% of distressed callers (TE), clinical teams validating culturally biased assessments affecting 28% of populations (SIV)
Analysis		
Technical evaluation	Evaluates: 31% CT-medication gap requiring beneficence adjustments (BN), familismo incompatible with individual consent – autonomy framework requires cultural adaptation over standardization (AIC), unknown accuracy for 24.2% minorities (JE), insufficient protection for 155 variables (PC), Spanish interpretation failures requiring redesign (TE), and 31% demographic disparities requiring population-specific calibration (SIV)	Evaluates: Beneficence framework determines 92.1% accuracy worth 2.3-s delays over faster alternatives (BN), autonomy conflicts in 847 capacity-impaired cases (AIC), justice implications for 23% accessibility barriers (JE), privacy-safety tension resolved through tiered encryption reducing delays to 1.5 s for urgent cases (PC), transparency failures in 66% of risk communications (TE), and cultural validation bias in 28% of populations (SIV)
Compliance assessment	Reviews: Audits finding no protocols for 31% accuracy disparities violating beneficence requirements (BN), state consent laws incompatible with Latino familismo creating autonomy violations requiring exemptions (AIC), HIPAA finds 75.8% White STAR*D sample violates diversity mandates creating justice-beneficence tension – framework guides decision to prioritize long-term equity over immediate implementation despite 6-month delay (JE), insufficient protection for 155 variables across multicultural sites (PC), FDA guidance lacking Spanish-language transparency creating compliance gaps (TE), and missing population-specific requirements for 31% disparities (SIV)	Reviews: Tension between rapid response (beneficence) and accurate assessment – framework accepts 2.3-s delays for safety (BN), regulatory exemptions for 847 capacity cases (AIC), compliance gaps in 23% accessibility (JE), HIPAA encryption conflicts with crisis response – framework prioritizes immediate safety with minimum privacy standards (PC), transparency violations in 66% of cases (TE), and cultural bias in 28% of validations (SIV)
Guideline review	Assesses: Ethics review finds no protocol for recommending 51% accurate medications over 82% therapy, prompting new beneficence standards (BN); legal review identifies HIPAA consent requirements conflicting with Latino family decision-making, requiring cultural autonomy policy revisions (AIC); IRB flags STAR*D inclusion criteria excluding 24% minorities, prompting diversity standards for validation (JE); audit finds HITECH compliance gaps in protecting 155 psychiatric variables, requiring enhanced privacy protocols (PC); documentation review notes the absence of Spanish-language AI explanations, mandating bilingual transparency policies (TE); and quality review identifies missing demographic performance tracking, requiring population-specific accuracy standards (SIV)	Assesses: Crisis intervention protocols against beneficence balance of accuracy versus speed – guidelines updated to accept 2.3-s delays for 92.1% precision (BN), emergency consent standards for 847 capacity-impaired cases (AIC), accessibility guidelines addressing 23% service barriers (JE), privacy frameworks modified to allow immediate access during suicide crises while maintaining encryption (PC), risk communication standards requiring simplified lexicon outputs (TE), cultural competency guidelines addressing 28% validation bias (SIV)
Decision-making		
Strategy development	Develops: Beneficence-guided thresholds accepting 51% sertraline versus 82% therapy based on safety analysis (BN), familismo-adapted consent strategies for 65% of Latino families (AIC), equity recruitment requiring 40% minority enrollment addressing 75.8% White bias (JE), encryption protocols for 155 variables across multicultural sites (PC), Spanish-language explanations targeting 66% comprehension failures (TE), and population-specific calibration addressing 31% demographic disparities (SIV)	Develops: Beneficence-guided thresholds accepting 2.3-s delays for 92.1% accuracy over speed-prioritized alternatives (BN), capacity-adapted consent strategies for 847 impaired cases (AIC), equity-focused accessibility improvements targeting 23% barrier reduction (JE), privacy-safety balanced protocols allowing immediate crisis access while maintaining data protection (PC), transparency enhancement reducing comprehension failures from 66% to target 20% (TE), and cultural validation strategies addressing 28% bias through population-specific calibration (SIV)
Implementation planning	Plans: Deployment balancing 51% medication accuracy against 82% therapy effectiveness with 6-week rollout (BN), bilingual consent protocols with family liaison procedures (AIC), 40% minority recruitment through community hiring (JE), VPN security for 155-variable database across 15 sites (PC), Spanish explanation videos reducing language barriers in 66% of cases (TE), and validation protocols addressing 31% disparities through population-specific testing (SIV)	Plans: Deployment timeline balancing 2.3-s processing delays against crisis response requirements with staged rollout (BN), consent protocol modifications for 847 capacity-varying cases with backup procedures (AIC), accessibility enhancement targeting 23% improvement through multilingual and technical upgrades (JE), security architecture redesign enabling immediate crisis access while preserving encryption for non-urgent data (PC), communication redesign reducing lexicon complexity for 66% comprehension improvement (TE), and cultural validation protocol addressing 28% bias through diverse testing (SIV)
Consensus building	Builds consensus: Stakeholder agreement accepting lower medication accuracy (51%) when therapy shows higher effectiveness (82%) among 25 psychiatrists (BN), familismo consent protocols balancing cultural autonomy with individual rights (AIC), 40% minority recruitment coalition addressing 75.8% White sample bias (JE), security-clinical agreement on encryption while accommodating multicultural confidentiality norms (PC), bilingual explanation requirements reducing 66% comprehension failures (TE), and population-specific validation standards addressing 31% demographic disparities (SIV)	Builds consensus: Stakeholder agreement on beneficence trade-off accepting 2.3-s delays for accuracy over speed (BN), collaborative protocols for 847 capacity-impaired cases balancing autonomy with protection needs (AIC), equity coalition supporting accessibility improvements affecting 23% of sessions (JE), security-clinical team agreement on privacy-safety balance allowing immediate access during crises (PC), transparency consensus on simplified explanations reducing 66% comprehension failures (TE), and cultural stakeholder alignment on validation improvements addressing 28% bias (SIV)
Implementation		
Clinical integration	Integrates: Epic EHR installation across 15 centers with 6-week rollout (BN), nurses collect family decision-maker names with bilingual consent forms (AIC), justice analysis determines deployment requires 40% Latino/African American recruitment with community liaisons (JE), VPN/two-factor authentication for psychiatric database (PC), Spanish explanation tablets with interpreter buttons (TE), and monthly ethnicity-based accuracy monitoring (SIV)	Activates: SR-BERT risk alerts with 2.3-s processing delays for 92.1% accuracy after rejecting faster but less reliable alternatives (BN), crisis chat capacity assessment protocols with modified consent procedures for 847 impaired decision-making cases (AIC), equitable response system addressing 23% accessibility barriers through multilingual interfaces and technical support (JE), chat security measures with privacy-safety balanced encryption reducing delays from 2.3 to 1.5 s while maintaining 256-bit protection (PC), lexicon-based risk communication system reducing comprehension failures from 66% to 23% (TE), and theory validation tracking across cultural populations addressing 28% baseline differences (SIV)
Staff training	Trains clinicians to explain 82% therapy versus 51% sertraline recommendations using standardized, patient-centered phrasing (BN); delivers role-play sessions practicing culturally adapted consent using family-based decision-making scenarios (AIC); provides bias training with real-world algorithm disparity cases and structured recognition exercises (JE); instructs on secure access and handling of psychiatric data using institutional privacy protocols (PC); teaches communication of AI outputs in bilingual formats supported by interpreter guidance (TE); and trains clinicians to interpret subgroup-specific model performance and identify accuracy gaps for review (SIV)	Trains on: SR-BERT assessment emphasizing balance between 92.1% accuracy and 2.3-s delays, with scenarios showing when speed versus accuracy takes priority (BN), crisis intervention capacity assessment for 847 cases involving impaired decision-making, including modified autonomy procedures (AIC), chat accessibility protocols for 23% of sessions affected by barriers, ensuring equitable service delivery (JE), data privacy procedures balancing encryption security with crisis response speed, teaching when immediate access overrides standard privacy protocols (PC), lexicon risk score interpretation reducing comprehension failures from 66% to 23% through simplified explanations (TE), and risk factor cultural validation recognizing 28% population differences in baseline presentations (SIV)
Performance monitoring	Monitors: 15% higher sertraline failures versus therapy revealing beneficence-autonomy conflict when safety monitoring shows family consent delays – framework guides balance prioritizing patient safety while respecting cultural processes (BN), 23 Latino families with familismo consent conflicts (AIC), 18% worse performance in African American patients (JE), 155 variables accessed 847 times with 12 unauthorized attempts (PC), 67% Spanish-speaking families do not understand explanations (TE), and 31% demographic disparities documented quarterly (SIV)	Monitors: SR-BERT response times showing 2.3-s delays reduced emergency response by 12% but prevented 67% more false positives – beneficence optimization confirmed (BN), crisis protocol adherence in 847 capacity-impaired cases with modified autonomy procedures reducing consent delays by 45% (AIC), service equity improvements reducing accessibility barriers from 23% to 8% through enhanced inclusion protocols (JE), chat security compliance showing 1.5-s encryption delays acceptable for nonurgent cases while immediate access maintained for high-risk situations (PC), lexicon communication with comprehension improvements from 34% to 77% following simplified explanations (TE), and prediction accuracy with bias reduction from 28% to 12% through adapted validation frameworks (SIV)
Review and reflection		
Outcome assessment	Assesses: Evaluation finds 25% improvement when clinicians override sertraline (51%) for therapy-suitable patients (82%) (BN), review documents of 18 Latino families completing familismo collective consent reducing delays 40% (AIC), analysis reveals 45% minority recruitment versus 24% baseline improving validation across populations (JE), audit shows 99.2% encryption compliance for 155 variables with zero breaches across 15 sites (PC), assessment finds 78% Spanish-speaking families understand explanations after interpreter implementation (TE), and validation documents demographic disparities reduced from 31% to 12% following population-specific calibration (SIV)	Assesses: SR-BERT intervention showing 2.3-s delays improving accuracy by 15% while reducing emergency response by 12% – framework analysis confirms net positive outcome (BN), capacity assessment protocols reducing consent conflicts from 847 to 234 cases through modified autonomy procedures (AIC), chat service improvements with accessibility barriers reduced from 23% to 8% benefiting underserved populations (JE), privacy protection resolving privacy-safety tension through tiered encryption reducing delays from 2.3 to 1.1 s (PC), lexicon communication with user comprehension improved from 34% to 77% through transparency enhancements (TE), and risk prediction accuracy with bias reduced from 28% to 12% through validation adaptations (SIV)
Performance evaluation	Analyses: Statistical analysis reveals 73% accuracy for high-confidence versus 45% low-confidence predictions requiring calibration (BN), evaluation shows 89% clinician satisfaction with therapy versus 52% with medications requiring improvements (AIC), fairness analysis discovers 28% accuracy gap between Latino and White patients requiring bias correction (JE), security assessment finds 94% protection for 155 variables but failures at six sites requiring upgrades (PC), comprehension testing shows 34% Spanish-speaking families understand outputs requiring interface redesign (TE), and methodology review finds validation adequate for whites but insufficient for 24% minorities requiring population-specific standards (SIV)	Analyses: Statistical analysis revealing 2.3-s delays achieved 92.1% accuracy versus 78% for faster alternatives, confirming framework-guided decision (BN), capacity assessment showing 89% successful consent modifications in 847 impaired cases (AIC), accessibility analysis documenting improvements from 23% to 8% barriers across diverse populations (JE), privacy-safety performance showing 1.5-s average delays maintaining security while enabling crisis response (PC), communication analysis revealing improvements from 34% to 77% comprehension through simplified lexicon (TE), and cultural validation showing bias reduction from 28% to 12% through population-specific calibration (SIV)
Framework refinement	Updates: Confidence thresholds raised 60% to 75% after discovering 23% false positives in sertraline recommendations (BN), protocols revised requiring family liaison when Latino patients request familismo decisions reducing delays 35% (AIC), standards updated requiring 40% minority enrollment after discovering bias in 75.8% White training (JE), protection enhanced with encryption after 12 unauthorized attempts on 155 variables (PC), frameworks redesigned with Spanish interfaces after 66% comprehension failures (TE), and validation expanded requiring population-specific testing after 31% demographic disparities (SIV)	Refines: Thresholds updated to 2.1-s delays for optimal accuracy-speed balance (BN), protocols enhanced for 15% improvement in 847 cases through refined autonomy procedures (AIC), standards elevated targeting 5% remaining barriers (JE), protocols optimized reducing delays to 1.2 s while maintaining crisis access (PC), frameworks improved targeting 85% comprehension through lexicon simplification (TE), validation expanded reducing bias below 8% through population-specific testing (SIV)

*Note*: Framework applications represent illustrative scenarios based on study limitations and known disparities in psychiatric AI to demonstrate concrete ethical decision-making processes. These examples are designed to show how the IEACP framework would guide decision-making in realistic clinical situations.

Ethical values: AIC, autonomy/informed consent; BN, beneficence/non-maleficence; JE, justice/equity; PC, privacy/confidentiality; TE, transparency/explainability; SIV, scientific integrity/validity.

Technical and clinical terms: APA, American Psychiatric Association; AUC, area under curve; CT, clinical trials; EHR, electronic health record; FDA, US Food and Drug Administration; HIPAA, Health Insurance Portability and Accountability Act; HITECH, Health Information Technology for Economic and Clinical Health Act; IRB, Institutional Review Board; SR-BERT, Suicide Risk–Bidirectional Encoder Representations From Transformers; STAR*D, Sequenced Treatment Alternatives to Relieve Depression; VPN, virtual private network.

The framework applications demonstrate the IEACP’s capacity to provide structured ethical guidance for computational psychiatry implementation. To contextualize this contribution within the existing landscape of AI ethics frameworks, we compare the IEACP with prominent approaches, including the WHO’s Ethics and Governance of AI for Health (World Health Organization, 2021), AI4People’s Ethical Framework (Floridi et al., 2018), and IEEE’s Ethically Aligned Design. Table 5 illustrates how the IEACP addresses gaps in existing frameworks by providing the first procedural approach specifically designed for computational psychiatry contexts.

**Table 5.** Ethical frameworks for AI: A comparative analysis featuring the IEACP

Dimension	Integrated ethical approach for computational psychiatry	WHO: Ethics and governance of AI for health	AI4People: Ethical framework for a good AI society	IEEE EAD: Ethically aligned design
Domain focus	Designed for computational psychiatry; integrates ethical principles with domain-specific implementation.	Digital and public health applications, especially in LMIC contexts.	Broad societal application of AI ethics in Europe.	Cross-sector focus on AI/AS systems with societal impact.
Core ethical values	Beneficence, autonomy, justice, privacy, transparency, and scientific integrity.	Autonomy, beneficence, safety, equity, explainability, and sustainability.	Beneficence, autonomy, justice, privacy, and transparency.	Human rights, wellbeing, accountability, transparency, and sustainability.
Framework structure	Five-stage procedural model (Identification, Analysis, Decision, Implementation, and Review) with embedded ethical processes.	Six high-level ethical principles guide policy and system design.	Five principles with 20 policy recommendations; strategic but nonoperational.	Ethical imperatives aligned with IEEE P7000 standards development.
Procedural orientation	Fully structured framework offering decision-making logic across all stages.	Lacks stepwise guidance; focuses on principles and outcomes.	Principles framed conceptually; no procedural schema.	Aspirational and value-based without decision process logic.
Clinical implementation	Offers concrete examples and practical integration guidance in mental health contexts.	Illustrates use cases; lacks domain-specific workflow detail.	Not designed for clinical translation or sector-specific tools.	Applicable to health tech contexts; lacks a direct implementation strategy.
Stakeholder integration	Emphasizes codesign, lived experience, and stakeholder mapping at each stage.	Acknowledged, but operational strategies are not defined.	Advocates for inclusion; no procedural mechanisms detailed.	Mentions participatory ethics and public consultation, but no defined engagement model.
Principle conflict resolution	Navigates trade-offs (e.g. privacy vs. transparency) through procedural arbitration at each stage.	Identifies ethical tensions but lacks resolution pathways.	Assumes principle alignment; does not address trade-offs.	Acknowledges value conflicts; provides no resolution protocol.
Novel contribution	Only framework offering procedural ethics tailored to computational psychiatry. Operationalizes principle application, stakeholder codesign, and ethical conflict resolution within real-world workflows.	Global normative guidance for AI in health, not domain- or discipline-specific.	Strategic vision for responsible AI in society lacks clinical focus.	Standard-setting vision; valuable for industry ethics but not grounded in healthcare implementation.

*Note*: This table compares four ethical frameworks for AI, emphasizing the distinctive procedural and domain-specific features of the Integrated Ethical Approach for Computational Psychiatry (IEACP). While these initiatives are commonly termed ‘frameworks’ in the AI ethics literature, they vary significantly in procedural specificity and implementation guidance, with the IEACP being the only approach providing structured decision-making processes for clinical implementation. Dimensions reflect core ethical values, structural orientation, clinical applicability, and stakeholder integration.

Abbreviations: AI, artificial intelligence; AI/AS, artificial intelligence and autonomous systems; IEEE EAD, Institute of Electrical and Electronics Engineers – Ethically Aligned Design; LMIC, low- and middle-income countries.

## Discussion

AI’s growing role in psychiatric care creates unresolved ethical challenges around patient autonomy, algorithmic bias, and clinical accountability. The IEACP framework addresses these challenges by integrating six core ethical values with a structured five-stage process. Unlike existing generalist guidelines for psychiatric ethics (Solanki et al., 2023; World Health Organization, 2021), the IEACP provides psychiatry-specific guidance, addressing the complexity of fluctuating mental states, clinician-patient dynamics, and the biopsychosocial model of care.

The framework’s dynamic ethical governance acknowledges that challenges in computational psychiatry evolve alongside AI advances and shifting psychiatric paradigms. (Barnett et al., 2018; Starke et al., 2021). For example, existing AI ethics frameworks often assume stable patient autonomy (Ploug & Holm, 2016; Vayena et al., 2018), whereas IEACP explicitly accounts for fluctuating decision-making capacities in psychiatric populations. Additionally, by incorporating stakeholder mapping and real-time performance monitoring, IEACP moves beyond static, principle-based ethical models to an adaptive framework that can accommodate emerging challenges, such as digital phenotyping, predictive psychiatry, and personalized treatment algorithms (Fusar-Poli et al., 2022; McCradden et al., 2023).

A key limitation of the IEACP framework is that it has not yet been empirically validated in real-world psychiatric AI applications. Although derived from a systematic analysis of 83 studies, the framework’s practical utility remains untested. However, similar principle-based ethical frameworks have been widely adopted based on their theoretical grounding rather than empirical validation (Floridi & Cowls, 2019; McCradden et al., 2023; Mittelstadt, 2019). The structured, principle-based nature of IEACP ensures its relevance in addressing AI ethics in psychiatry, even in the absence of direct validation.

To bridge this gap, future research should pilot-test the IEACP framework in psychiatric AI implementation. A mixed-method evaluation could involve (1) clinician decision-making studies assessing its applicability in guiding AI-assisted psychiatric interventions, (2) stakeholder engagement studies incorporating patient and clinician perspectives, and (3) real-world validation through case applications in clinical psychiatry. Establishing an iterative refinement process through empirical testing would enhance the framework’s adaptability and ensure alignment with evolving computational psychiatry challenges. Such research will be critical in advancing the IEACP from a theoretically grounded model to an empirically validated tool for ethical AI integration in psychiatric practice.

## Conclusion

Computational psychiatry requires ethical approaches that balance innovation with human-centered care. The IEACP framework offers a systematic method for addressing ethical challenges in AI-driven psychiatric applications. Unlike retrospective evaluations, it embeds ethical considerations into AI development, reducing oversight risks and aligning with bioethical principles. Its design supports immediate use and future adaptation. Pilot studies and refinement will enhance its applicability. IEACP may catalyze domain-specific ethical frameworks that preserve psychiatry’s human foundations.

## Supporting information

Putica et al. supplementary material Putica et al. supplementary material

## Supplementary material

The supplementary material for this article can be found at http://doi.org/10.1017/S0033291725101311.
