# Supplementary material for: Ethical decision-making for AI in mental health: the Integrated Ethical Approach for Computational Psychiatry (IEACP) framework
Source: Psychol Med. 2025 Jul 24;55:e213. doi: 10.1017/S0033291725101311 (PMC12315656; doi:10.1017/S0033291725101311)
Supplement: Putica et al. supplementary material [file S0033291725101311sup001.zip › Supplementary Material.docx]

**Supplementary Material: Literature Search Strategy and Study Selection**

***Literature Search Strategy***

We conducted a systematic search of peer-reviewed literature using three major databases: Scopus, PubMed, and Web of Science from inception to December 17, 2024. The search strategy was designed to capture the intersection of computational psychiatry, ethics, and decision-making frameworks. Our search string was: ("computational psychiatry" OR "digital psychiatry" OR "precision psychiatry" OR "AI in psychiatry" OR "machine learning in psychiatry") AND (ethics OR ethical OR bioethics OR "moral considerations" OR "ethical implications" OR "ethical challenges") AND ("decision-making" OR "clinical decision" OR "framework" OR "guideline" OR "approach").

***Study Selection***

Articles were managed using Covidence systematic review software (Veritas Health Innovation, Melbourne, Australia). The selection process followed a systematic two-stage approach: initial screening of titles and abstracts followed by full-text review. One reviewer conducted the screening process, with regular consultations with the research team for articles where inclusion decisions were unclear. This approach allowed for consistent application of the inclusion and exclusion criteria while maintaining efficiency appropriate for framework development. Screening was conducted by a single reviewer, consistent with conceptual framework development methods where interpretive synthesis is central (Jabareen, 2009), and with flexible scoping review frameworks that allow this where justified by scope and feasibility (Arksey & O’Malley, 2005). Ambiguities were resolved through team discussion.

Inclusion criteria encompassed: (1) peer-reviewed articles in English, (2) studies focusing on or discussing ethical issues specifically in computational psychiatry or related fields, (3) papers discussing ethical frameworks or decision-making processes in the context of AI/ML applications in psychiatry, and (4) article types including original research, systematic reviews/meta-analyses, theoretical papers, implementation case studies, expert consensus statements, policy papers and framework development articles. Exclusion criteria were: (1) non-peer reviewed literature, (2) papers without substantive focus on ethical considerations, (3) articles discussing general AI ethics without specific application to psychiatry, (4) opinion pieces without substantive ethical analysis, (5) papers focused solely on technical implementation without ethical considerations, (6) non-English language articles, and (7) gray literature.

The initial database search yielded 1213 records. After removal of duplicates, 1173 articles underwent title and abstract screening. Following full-text review, 83 studies met all inclusion criteria and were included in the final analysis. A PRISMA flow diagram detailing the complete selection process is provided in Figure S-1 below.

**Figure S-1**

PRISMA flow diagram of study selection.


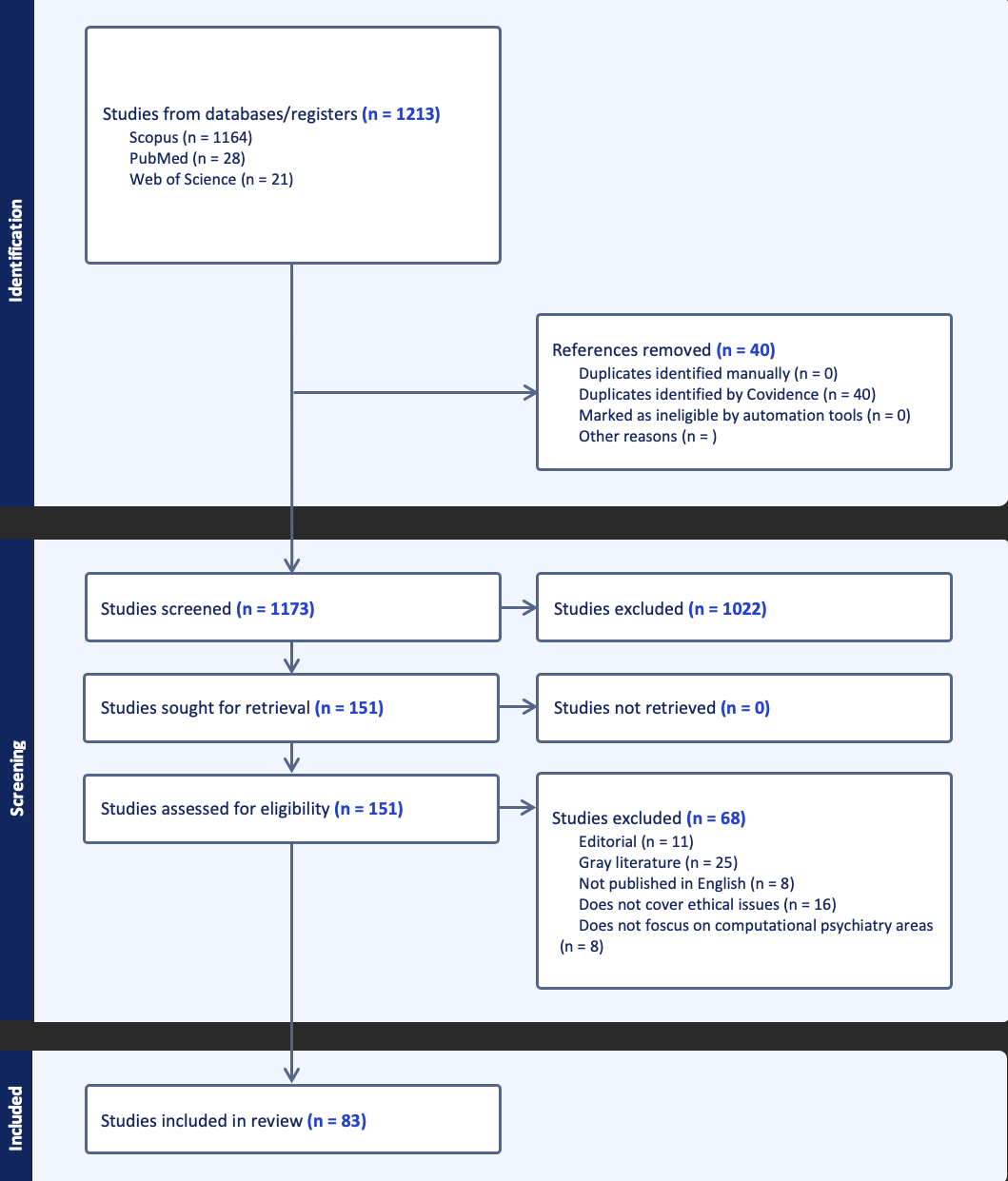


**Data Extraction and Synthesis**

Data extraction followed a structured approach using a pre-designed form capturing five key domains: Study ID, Study Type/Focus, Ethical Challenges Identified, Ethical Values Identified/Addressed, and Implementation Context. Data extraction was conducted by a primary reviewer (AP), with regular validation checks through research team discussions to ensure consistency and comprehensiveness of the extraction process. The complete data extraction table is provided in Supplementary Table S-1.

***Framework Development Procedure***

We employed systematic search methods that prioritized conceptual synthesis over traditional intervention reviews (Jabareen, 2009; Kallio, Pietilä, Johnson, & Kangasniemi, 2016). We adapted PRISMA guidelines (Page et al., 2021) to fit framework development goals, excluding statistical synthesis given conceptual framework focus. Our methodology focused on identifying and integrating ethical considerations in computational psychiatry to develop a practical framework, aligning with established approaches in health care and bioethics (Arksey & O’Malley, 2005; Levac, Colquhoun, & O’Brien, 2010).

The IEACP framework emerged via iterative synthesis consistent with conceptual framework development (Jabareen, 2009). This method involves iterative reading, identification, categorization, and integration of concepts drawn from a heterogeneous literature base, with the goal of constructing a coherent theoretical model. Unlike thematic analysis, which is typically used in primary qualitative research to identify semantic or latent patterns in participant data, interpretive synthesis is designed for secondary conceptual work, aiming to generate new theoretical constructs. Similarly, the approach differs from philosophical conceptual analysis, which focuses on clarifying the necessary and sufficient conditions of concepts. Instead, this framework development process relied on repeated rounds of literature engagement to extract, cluster, and refine ethically salient concepts and procedural elements relevant to computational psychiatry. These concepts were then organized into five procedural stages, each with three implementation processes, and aligned with six core ethical values identified across the literature (see Supplementary Table S-1 and S-2). The final framework was refined through interdisciplinary review and consensus across experts in psychiatry, digital mental health, ethics, and lived experience.

**References**

Arksey, H., & and O’Malley, L. (2005). Scoping studies: Towards a methodological framework. *International Journal of Social Research Methodology*, *8*(1), 19–32. https://doi.org/10.1080/1364557032000119616

Arksey, H., & O’Malley, L. (2005). Scoping studies: Towards a methodological framework. *International Journal of Social Research Methodology*, *8*(1), 19–32. https://doi.org/10.1080/1364557032000119616

Jabareen, Y. (2009). Building a Conceptual Framework: Philosophy, Definitions, and Procedure. *International Journal of Qualitative Methods*, *8*(4), 49–62. https://doi.org/10.1177/160940690900800406

Kallio, H., Pietilä, A.-M., Johnson, M., & Kangasniemi, M. (2016). Systematic methodological review: Developing a framework for a qualitative semi-structured interview guide. *Journal of Advanced Nursing*, *72*(12), 2954–2965. https://doi.org/10.1111/jan.13031

Levac, D., Colquhoun, H., & O’Brien, K. K. (2010). Scoping studies: Advancing the methodology. *Implementation Science*, *5*(1), 69. https://doi.org/10.1186/1748-5908-5-69

Page, M. J., McKenzie, J. E., Bossuyt, P. M., Boutron, I., Hoffmann, T. C., Mulrow, C. D., … Brennan, S. E. (2021). The PRISMA 2020 statement: An updated guideline for reporting systematic reviews. *International Journal of Surgery*, *88*, 105906. Retrieved from https://www.sciencedirect.com/science/article/pii/S1743919121000406
