# Supplementary material for: Ethical decision-making for AI in mental health: the Integrated Ethical Approach for Computational Psychiatry (IEACP) framework
Source: Psychol Med. 2025 Jul 24;55:e213. doi: 10.1017/S0033291725101311 (PMC12315656; doi:10.1017/S0033291725101311)
Supplement: Putica et al. supplementary material [file S0033291725101311sup001.zip › Supplementary Table 2_PM.docx]

**Supplementary Table S-2**

Frequency Distribution of Core Ethical Values in Computational Psychiatry Literature (N=83)

| Ethical Value | Number of Studies | Percentage | Common Implementation Contexts | Contributing Studies |
| --- | --- | --- | --- | --- |
| Privacy & Confidentiality | 70 | 82.35% | Digital phenotyping (65.10%)  Electronic health records (58.10%)  Remote monitoring (52.30%) | (Ahmed and Hens 2022; Arbanas 2024; Avula and Amalakanti 2024; Ball, Kalinowski, and Williams 2020; Bentley et al. 2021; Bertl, Ross, and Draheim 2022; Briganti 2023; Chekroud et al. 2021; I. Chen et al. 2022; Z. S. Chen et al. 2022; Clarke, Foltz, and Garrard 2020; D’Alfonso 2020; Davidson 2022; Diaz-Asper et al. 2024; D’Souza et al. 2024; Dube et al. 2024; Dwyer and Koutsouleris 2022; Espejo, Reiner, and Wenzinger 2023; Faissner et al. 2024; Fardouly, Crosby, and Sukunesan 2022; Farmer et al. 2024; Fusar-Poli et al. 2022; Gillett and Saunders 2019; Gooding and Kariotis 2021; Graham et al. 2019; Gültekin and Şahin 2024; Hagendorff 2021; Hart et al. 2022; Hurley et al. 2024; Islam et al. 2024; Jacobson et al. 2020; Jin et al. 2023; Kappen, Vanderhasselt, and Slavich 2023; Kirtley et al. 2022; Kleine et al. 2023; Kolding et al. 2024; Koutsouleris et al. 2022; Leung 2023; Manchia et al. 2020; Mazor et al. 2023; McCradden, Hui, and Buchman 2023; Monaco et al. 2024; Monosov et al. 2024; Monteith et al. 2022, 2023; Morley et al. 2020; Murray et al. 2021; Ortiz and Mulsant 2024; Oudin et al. 2023; Parziale and Mascalzoni 2022; Pavlopoulos, Rachiotis, and Maglogiannis 2024; Rahul et al. 2024; Rocheteau 2023; Saheb, Saheb, and Carpenter 2021; Shen et al. 2022; Singhal et al. 2024; Smith et al. 2023; Starke et al. 2021; Stein and Prost 2024; Straw 2021; Sultan, Scholz, and van den Bos 2023; Terra et al. 2023; Thieme, Belgrave, and Doherty 2020; Torous et al. 2021; Upreti et al. 2024; Vale 2024; Wang et al. 2024; Wiese and Friston 2022; Wouters et al. 2024; Wray et al. 2021; Zhang et al. 2023; Zidaru, Morrow, and Stockley 2021) |
| Transparency & Explainability | 63 | 74.12% | Clinical decision support (69.80%)  Risk prediction (62.80%)  Treatment selection (55.80%) | (Avula and Amalakanti 2024; Ball et al. 2020; Bertl et al. 2022; Briganti 2023; Chekroud et al. 2021; Z. S. Chen et al. 2022; D’Alfonso 2020; Diaz-Asper et al. 2024; D’Souza et al. 2024; Dube et al. 2024; Espejo et al. 2023; Faissner et al. 2024; Farmer et al. 2024; Fisher 2024; Fusar-Poli et al. 2022; Graham et al. 2019; Gültekin and Şahin 2024; Hagendorff 2021; Heinrichs and Eickhoff 2020; Hurley et al. 2024; Jin et al. 2023; Kirtley et al. 2022; Kleine et al. 2023; Kolding et al. 2024; Koutsouleris et al. 2022; Levkovich, Shinan-Altman, and Elyoseph 2024; Lewis et al. 2024; Manchia et al. 2020; Mazor et al. 2023; McCradden et al. 2023; Monaco et al. 2024; Monteith et al. 2022, 2023; Morley et al. 2020; Ortiz and Mulsant 2024; Ostojic et al. 2024; Oudin et al. 2023; Pavlopoulos et al. 2024; Rahul et al. 2024; Rocheteau 2023; Saheb et al. 2021; Sahin et al. 2024; Singhal et al. 2024; Skorburg, O’Doherty, and Friesen 2024; Smith et al. 2023:202; Starke et al. 2021; Straw 2021; Terra et al. 2023; Thieme et al. 2020; Torous et al. 2021; Vale 2024; Wang et al. 2024; Wiese and Friston 2022; Wouters et al. 2024; Wray et al. 2021; Zhang et al. 2023; Zidaru et al. 2021) |
| Justice & Equity | 55 | 64.71% | Diagnostic systems (55.80%)  Treatment allocation (51.20%)  Service delivery (47.70%) | (Arbanas 2024; Avula and Amalakanti 2024; Ball et al. 2020; Bertl et al. 2022; Briganti 2023; I. Chen et al. 2022; Z. S. Chen et al. 2022; D’Souza et al. 2024; Dube et al. 2024; Dwyer and Koutsouleris 2022; Espejo et al. 2023; Faissner et al. 2024; Farmer et al. 2024; Fisher 2024; Fusar-Poli et al. 2022; Galderisi et al. 2024; Gooding and Kariotis 2021; Graham et al. 2019; Gültekin and Şahin 2024; Hagendorff 2021; Islam et al. 2024; Jin et al. 2023; Kleine et al. 2023; Koutsouleris et al. 2022; Levkovich et al. 2024; Lewis et al. 2024; Mazor et al. 2023; McCradden et al. 2023; Monaco et al. 2024; Monteith et al. 2022, 2023; Morley et al. 2020; Murray et al. 2021; Oudin et al. 2023; Pavlopoulos et al. 2024; Rahul et al. 2024; Rocheteau 2023; Saheb et al. 2021; Sahin et al. 2024; Singhal et al. 2024; Skorburg et al. 2024; Smith et al. 2023; Starke et al. 2021; Stein and Prost 2024; Terra et al. 2023; Thieme et al. 2020; Torous et al. 2021; Vale 2024; Wang et al. 2024; Wiese and Friston 2022; Wouters et al. 2024; Wray et al. 2021; Zhang et al. 2023; Zidaru et al. 2021) |
| Beneficence & Non-maleficence | 50 | 58.82% | Patient safety protocols (54.7%)  Risk-benefit assessment (52.3%)  Intervention timing (47.7%) | (Ball et al. 2020; Bentley et al. 2021; Briganti 2023; Z. S. Chen et al. 2022; D’Souza et al. 2024; Dube et al. 2024; Dwyer and Koutsouleris 2022; Espejo et al. 2023; Farmer et al. 2024; Fisher 2024; Fusar-Poli et al. 2022; Galderisi et al. 2024; Gillett and Saunders 2019; Graham et al. 2019; Gültekin and Şahin 2024; Hagendorff 2021; Hurley et al. 2024; Jacobson et al. 2020; Jin et al. 2023; Kirtley et al. 2022; Kolding et al. 2024; Koutsouleris et al. 2022; Leung 2023; Levkovich et al. 2024; Manchia et al. 2020; Mazor et al. 2023; McCradden et al. 2023; Monaco et al. 2024; Monteith et al. 2022, 2023; Murray et al. 2021; Ortiz and Mulsant 2024; Oudin et al. 2023; Pavlopoulos et al. 2024; Rocheteau 2023; Saheb et al. 2021; Singhal et al. 2024; Smith et al. 2023; Starke et al. 2021; Straw 2021; Terra et al. 2023; Thieme et al. 2020; Torous et al. 2021; Vale 2024; Wang et al. 2024; Wiese and Friston 2022; Wouters et al. 2024; Wray et al. 2021; Zhang et al. 2023; Zidaru et al. 2021) |
| Autonomy & Informed Consent | 47 | 55.29% | Treatment planning (48.80%)  Data collection consent (44.20%)  Risk assessment (41.90%) | (Ahmed and Hens 2022; Arbanas 2024; Ball et al. 2020; Z. S. Chen et al. 2022; Davidson 2022; D’Souza et al. 2024; Faissner et al. 2024; Farmer et al. 2024; Fisher 2024; Fusar-Poli et al. 2022; Galderisi et al. 2024; Gillett and Saunders 2019; Graham et al. 2019; Gültekin and Şahin 2024; Hagendorff 2021; Hurley et al. 2024; Jacobson et al. 2020; Jin et al. 2023; Kappen et al. 2023; Lewis et al. 2024; Manchia et al. 2020; McCradden et al. 2023; Monaco et al. 2024; Murray et al. 2021; Ortiz and Mulsant 2024; Oudin et al. 2023; Pavlopoulos et al. 2024; Rocheteau 2023; Saheb et al. 2021; Shen et al. 2022; Skorburg et al. 2024; Smith et al. 2023; Stein and Prost 2024; Straw 2021; Sultan et al. 2023; Terra et al. 2023; Thieme et al. 2020; Torous et al. 2021; Vale 2024; Wang et al. 2024; Wiese and Friston 2022; Wouters et al. 2024; Wray et al. 2021; Zidaru et al. 2021) |
| Scientific Integrity & Validity | 44 | 51.76% | Model validation (48.80%)  Clinical translation (44.20%)  Quality assessment (41.90%) | (Ahmed and Hens 2022; Ball et al. 2020; Bentley et al. 2021; Bertl et al. 2022; Chekroud et al. 2021; Z. S. Chen et al. 2022; Clarke et al. 2020; Dikaios et al. 2023; Dwyer and Koutsouleris 2022; Espejo et al. 2023; Fusar-Poli et al. 2022; Hagendorff 2021; Hart et al. 2022; Heinrichs and Eickhoff 2020; Islam et al. 2024; Kirtley et al. 2022; Lewis et al. 2024; Mazor et al. 2023; McCradden et al. 2023; Monosov et al. 2024; Monteith et al. 2022, 2023; Murray et al. 2021; Ortiz and Mulsant 2024; Ostojic et al. 2024; Oudin et al. 2023; Rahul et al. 2024; Sahin et al. 2024; Smith et al. 2023; Starke et al. 2021; Stein and Prost 2024; Straw 2021; Tabb and Lemoine 2021; Tejavibulya et al. 2022; Terra et al. 2023; Thieme et al. 2020; Torous et al. 2021; Wang et al. 2024; Wiese and Friston 2022; Wouters et al. 2024; Wray et al. 2021) |

Note. Percentages indicate proportion of studies addressing each ethical value and implementation context. Implementation contexts represent the three most frequently documented applications for each ethical value based on systematic coding of the extraction data. Percentages and frequencies greater than 100 due to multiple values being endorsed across studies.

**References**

Ahmed, Eman, and Kristien Hens. 2022. “Microbiome in Precision Psychiatry: An Overview of the Ethical Challenges Regarding Microbiome Big Data and Microbiome-Based Interventions.” *AJOB Neuroscience* 13(4):270–86. doi: 10.1080/21507740.2021.1958096.

Arbanas, Goran. 2024. “ChatGPT and Other Chatbots in Psychiatry.” *Archives of Psychiatry Research : An International Journal of Psychiatry and Related Sciences* 60.(2.):137–42. doi: 10.20471/june.2024.60.02.07.

Avula, Vijaya Chandra Reddy, and Sridhar Amalakanti. 2024. “Artificial Intelligence in Psychiatry, Present Trends, and Challenges: An Updated Review.” *Archives of Mental Health* 25(1).

Ball, Tali M., Agnieszka Kalinowski, and Leanne M. Williams. 2020. “Ethical Implementation of Precision Psychiatry.” *Personalized Medicine in Psychiatry* 19–20:100046. doi: 10.1016/j.pmip.2019.05.003.

Bentley, Kate H., Joseph S. Maimone, Erin N. Kilbury, Marshall S. Tate, Hannah Wisniewski, M. Taylor Levine, Regina Roberg, John B. Torous, Matthew K. Nock, and Evan M. Kleiman. 2021. “Practices for Monitoring and Responding to Incoming Data on Self-Injurious Thoughts and Behaviors in Intensive Longitudinal Studies: A Systematic Review.” *Clinical Psychology Review* 90:102098. doi: 10.1016/j.cpr.2021.102098.

Bertl, Markus, Peeter Ross, and Dirk Draheim. 2022. “A Survey on AI and Decision Support Systems in Psychiatry – Uncovering a Dilemma.” *Expert Systems with Applications* 202:117464. doi: 10.1016/j.eswa.2022.117464.

Briganti, Giovanni. 2023. “Artificial Intelligence in Psychiatry.” *Psychiatria Danubina* 35(Suppl 2):15–19.

Chekroud, Adam M., Julia Bondar, Jaime Delgadillo, Gavin Doherty, Akash Wasil, Marjolein Fokkema, Zachary Cohen, Danielle Belgrave, Robert DeRubeis, Raquel Iniesta, Dominic Dwyer, and Karmel Choi. 2021. “The Promise of Machine Learning in Predicting Treatment Outcomes in Psychiatry.” *World Psychiatry* 20(2):154–70. doi: 10.1002/wps.20882.

Chen, IM, YY Chen, SC Liao, and YH Lin. 2022. “Development of Digital Biomarkers of Mental Illness via Mobile Apps for Personalized Treatment and Diagnosis.” *JOURNAL OF PERSONALIZED MEDICINE* 12(6). doi: 10.3390/jpm12060936.

Chen, Zhe Sage, Prathamesh (Param) Kulkarni, Isaac R. Galatzer-Levy, Benedetta Bigio, Carla Nasca, and Yu Zhang. 2022. “Modern Views of Machine Learning for Precision Psychiatry.” *Patterns* 3(11). doi: 10.1016/j.patter.2022.100602.

Clarke, Natasha, Peter Foltz, and Peter Garrard. 2020. “How to Do Things with (Thousands of) Words: Computational Approaches to Discourse Analysis in Alzheimer’s Disease.” *Cortex* 129:446–63. doi: 10.1016/j.cortex.2020.05.001.

D’Alfonso, Simon. 2020. “AI in Mental Health.” *Cyberpsychology* 36:112–17. doi: 10.1016/j.copsyc.2020.04.005.

Davidson, Brittany I. 2022. “The Crossroads of Digital Phenotyping.” *General Hospital Psychiatry* 74:126–32. doi: 10.1016/j.genhosppsych.2020.11.009.

Diaz-Asper, Catherine, Mathias K. Hauglid, Chelsea Chandler, Alex S. Cohen, Peter W. Foltz, and Brita Elvevåg. 2024. “A Framework for Language Technologies in Behavioral Research and Clinical Applications: Ethical Challenges, Implications, and Solutions.” *The American Psychologist* 79(1):79–91. doi: 10.1037/amp0001195.

Dikaios, Katerina, Sheri Rempel, Sri Harsha Dumpala, Sageev Oore, Michael Kiefte, and Rudolf Uher. 2023. “Applications of Speech Analysis in Psychiatry.” *Harvard Review of Psychiatry* 31(1).

D’Souza, RF, M. Mathew, S. Amanullah, JE Thornton, V. Mishra, E. Mohandas, PL Palatty, and KM Surapaneni. 2024. “Navigating Merits and Limits on the Current Perspectives and Ethical Challenges in the Utilization of Artificial Intelligence in Psychiatry - An Exploratory Mixed Methods Study.” *ASIAN JOURNAL OF PSYCHIATRY* 97. doi: 10.1016/j.ajp.2024.104067.

Dube, AR, AJH Ambrose, G. Velez, and M. Jadhav. 2024. “Real Concerns, Artificial Intelligence: Reality Testing for Psychiatrists.” *INTERNATIONAL REVIEW OF PSYCHIATRY*. doi: 10.1080/09540261.2024.2363374.

Dwyer, Dominic, and Nikolaos Koutsouleris. 2022. “Annual Research Review: Translational Machine Learning for Child and Adolescent Psychiatry.” *Journal of Child Psychology and Psychiatry* 63(4):421–43. doi: 10.1111/jcpp.13545.

Espejo, Gemma, Wade Reiner, and Michael Wenzinger. 2023. “Exploring the Role of Artificial Intelligence in Mental Healthcare: Progress, Pitfalls, and Promises.” *Cureus* 15(9):e44748. doi: 10.7759/cureus.44748.

Faissner, Mirjam, Eva Kuhn, Regina Müller, and Sebastian Laacke. 2024. “Detecting Your Depression with Your Smartphone? – An Ethical Analysis of Epistemic Injustice in Passive Self-Tracking Apps.” *Ethics and Information Technology* 26(2):28. doi: 10.1007/s10676-024-09765-7.

Fardouly, Jasmine, Ross D. Crosby, and Suku Sukunesan. 2022. “Potential Benefits and Limitations of Machine Learning in the Field of Eating Disorders: Current Research and Future Directions.” *Journal of Eating Disorders* 10(1):66. doi: 10.1186/s40337-022-00581-2.

Farmer, Ryan L., Adam B. Lockwood, Anisa Goforth, and Christopher Thomas. 2024. “Artificial Intelligence in Practice: Opportunities, Challenges, and Ethical Considerations.” *Professional Psychology: Research and Practice* No Pagination Specified-No Pagination Specified. doi: 10.1037/pro0000595.

Fisher, CE. 2024. “The Real Ethical Issues with AI for Clinical Psychiatry.” *INTERNATIONAL REVIEW OF PSYCHIATRY*. doi: 10.1080/09540261.2024.2376575.

Fusar-Poli, Paolo, Mirko Manchia, Nikolaos Koutsouleris, David Leslie, Christiane Woopen, Monica E. Calkins, Michael Dunn, Christophe Le Tourneau, Miia Mannikko, Tineke Mollema, Dominic Oliver, Marcella Rietschel, Eva Z. Reininghaus, Alessio Squassina, Lucia Valmaggia, Lars Vedel Kessing, Eduard Vieta, Christoph U. Correll, Celso Arango, and Ole A. Andreassen. 2022. “Ethical Considerations for Precision Psychiatry: A Roadmap for Research and Clinical Practice.” *European Neuropsychopharmacology* 63:17–34. doi: 10.1016/j.euroneuro.2022.08.001.

Galderisi, Silvana, Paul S. Appelbaum, Neeraj Gill, Piers Gooding, Helen Herrman, Antonio Melillo, Keris Myrick, Soumitra Pathare, Martha Savage, George Szmukler, and John Torous. 2024. “Ethical Challenges in Contemporary Psychiatry: An Overview and an Appraisal of Possible Strategies and Research Needs.” *World Psychiatry* 23(3):364–86. doi: 10.1002/wps.21230.

Gillett, George, and Kate E. A. Saunders. 2019. “Remote Monitoring for Understanding Mechanisms and Prediction in Psychiatry.” *Current Behavioral Neuroscience Reports* 6(2):51–56. doi: 10.1007/s40473-019-00176-3.

Gooding, Piers, and Timothy Kariotis. 2021. “Ethics and Law in Research on Algorithmic and Data-Driven Technology in Mental Health Care: Scoping Review.” *JMIR Ment Health* 8(6):e24668. doi: 10.2196/24668.

Graham, Sarah, Colin Depp, Ellen E. Lee, Camille Nebeker, Xin Tu, Ho-Cheol Kim, and Dilip V. Jeste. 2019. “Artificial Intelligence for Mental Health and Mental Illnesses: An Overview.” *Current Psychiatry Reports* 21(11):116. doi: 10.1007/s11920-019-1094-0.

Gültekin, Mücahit, and Meryem Şahin. 2024. “The Use of Artificial Intelligence in Mental Health Services in Turkey: What Do Mental Health Professionals Think?” *Cyberpsychology: Journal of Psychosocial Research on Cyberspace* 18(1). doi: 10.5817/CP2024-1-6.

Hagendorff, Thilo. 2021. “Linking Human And Machine Behavior: A New Approach to Evaluate Training Data Quality for Beneficial Machine Learning.” *Minds and Machines* 31(4):563–93. doi: 10.1007/s11023-021-09573-8.

Hart, Alexander, Dorota Reis, Elisabeth Prestele, and Nicholas C. Jacobson. 2022. “Using Smartphone Sensor Paradata and Personalized Machine Learning Models to Infer Participants’ Well-Being: Ecological Momentary Assessment.” *J Med Internet Res* 24(4):e34015. doi: 10.2196/34015.

Heinrichs, Bert, and Simon B. Eickhoff. 2020. “Your Evidence? Machine Learning Algorithms for Medical Diagnosis and Prediction.” *Human Brain Mapping* 41(6):1435–44. doi: 10.1002/hbm.24886.

Hurley, Meghan E., Anika Sonig, John Herrington, Eric A. Storch, Gabriel Lázaro-Muñoz, Jennifer Blumenthal-Barby, and Kristin Kostick-Quenet. 2024. “Ethical Considerations for Integrating Multimodal Computer Perception and Neurotechnology.” *Frontiers in Human Neuroscience* 18. doi: 10.3389/fnhum.2024.1332451.

Islam, Md. Monirul, Shahriar Hassan, Sharmin Akter, Ferdaus Anam Jibon, and Md. Sahidullah. 2024. “A Comprehensive Review of Predictive Analytics Models for Mental Illness Using Machine Learning Algorithms.” *Healthcare Analytics* 6:100350. doi: 10.1016/j.health.2024.100350.

Jacobson, Nicholas C., Kate H. Bentley, Ashley Walton, Shirley B. Wang, Rebecca G. Fortgang, Alexander J. Millner, Garth Coombs, Alexandra M. Rodman, and Daniel D. L. Coppersmith. 2020. “Ethical Dilemmas Posed by Mobile Health and Machine Learning in Psychiatry Research.” *Bulletin of the World Health Organization* 98(4):270–76. doi: 10.2471/BLT.19.237107.

Jin, Kevin W., Qiwei Li, Yang Xie, and Guanghua Xiao. 2023. “Artificial Intelligence in Mental Healthcare: An Overview and Future Perspectives.” *British Journal of Radiology* 96(1150):20230213. doi: 10.1259/bjr.20230213.

Kappen, Mitchel, Marie-Anne Vanderhasselt, and George M. Slavich. 2023. “Speech as a Promising Biosignal in Precision Psychiatry.” *Neuroscience & Biobehavioral Reviews* 148:105121. doi: 10.1016/j.neubiorev.2023.105121.

Kirtley, Olivia J., Kasper van Mens, Mark Hoogendoorn, Navneet Kapur, and Derek de Beurs. 2022. “Translating Promise into Practice: A Review of Machine Learning in Suicide Research and Prevention.” *The Lancet Psychiatry* 9(3):243–52. doi: 10.1016/S2215-0366(21)00254-6.

Kleine, Anne-Kathrin, Eva Lermer, Julia Cecil, Anna Heinrich, and Susanne Gaube. 2023. “Advancing Mental Health Care with AI-Enabled Precision Psychiatry Tools: A Patent Review.” *Computers in Human Behavior Reports* 12:100322. doi: 10.1016/j.chbr.2023.100322.

Kolding, Sara, Robert M. Lundin, Lasse Hansen, and Søren Dinesen Østergaard. 2024. “Use of Generative Artificial Intelligence (AI) in Psychiatry and Mental Health Care: A Systematic Review.” *Acta Neuropsychiatrica* 1–14. doi: 10.1017/neu.2024.50.

Koutsouleris, Nikolaos, Tobias U. Hauser, Vasilisa Skvortsova, and Munmun De Choudhury. 2022. “From Promise to Practice: Towards the Realisation of AI-Informed Mental Health Care.” *The Lancet Digital Health* 4(11):e829–40. doi: 10.1016/S2589-7500(22)00153-4.

Leung, Ricky. 2023. “Using AI–ML to Augment the Capabilities of Social Media for Telehealth and Remote Patient Monitoring.” *Healthcare* 11(12). doi: 10.3390/healthcare11121704.

Levkovich, Inbar, Shiri Shinan-Altman, and Zohar Elyoseph. 2024. “Can Large Language Models Be Sensitive to Culture Suicide Risk Assessment?”

Lewis, Anna C. F., Rex L. Chisholm, John J. Connolly, Edward D. Esplin, Joe Glessner, Adam Gordon, Robert C. Green, Hakon Hakonarson, Margaret Harr, Ingrid A. Holm, Gail P. Jarvik, Elizabeth Karlson, Eimear E. Kenny, Leah Kottyan, Niall Lennon, Jodell E. Linder, Yuan Luo, Lisa J. Martin, Emma Perez, Megan J. Puckelwartz, Laura J. Rasmussen-Torvik, Maya Sabatello, Richard R. Sharp, Jordan W. Smoller, Rene Sterling, Shannon Terek, Wei-Qi Wei, and Stephanie M. Fullerton. 2024. “Managing Differential Performance of Polygenic Risk Scores across Groups: Real-World Experience of the eMERGE Network.” *The American Journal of Human Genetics* 111(6):999–1005. doi: 10.1016/j.ajhg.2024.04.005.

Manchia, Mirko, Claudia Pisanu, Alessio Squassina, and Bernardo Carpiniello. 2020. “Challenges and Future Prospects of Precision Medicine in Psychiatry.” *Pharmacogenomics and Personalized Medicine* 13(null):127–40. doi: 10.2147/PGPM.S198225.

Mazor, Matan, Simon Brown, Anna Ciaunica, Athena Demertzi, Johannes Fahrenfort, Nathan Faivre, Jolien C. Francken, Dominique Lamy, Bigna Lenggenhager, Michael Moutoussis, Marie-Christine Nizzi, Roy Salomon, David Soto, Timo Stein, and Nitzan Lubianiker. 2023. “The Scientific Study of Consciousness Cannot and Should Not Be Morally Neutral.” *Perspectives on Psychological Science* 18(3):535–43. doi: 10.1177/17456916221110222.

McCradden, Melissa, Katrina Hui, and Daniel Z. Buchman. 2023. “Evidence, Ethics and the Promise of Artificial Intelligence in Psychiatry.” *Journal of Medical Ethics* 49(8):573. doi: 10.1136/jme-2022-108447.

Monaco, Francesco, Annarita Vignapiano, Martina Piacente, Claudio Pagano, Carlo Mancuso, Luca Steardo, Alessandra Marenna, Federica Farina, Gianvito Petrillo, Stefano Leo, Emanuela Ferrara, Stefania Palermo, Vassilis Martiadis, Marco Solmi, Alessio Maria Monteleone, Alessio Fasano, and Giulio Corrivetti. 2024. “An Advanced Artificial Intelligence Platform for a Personalised Treatment of Eating Disorders.” *Frontiers in Psychiatry* 15. doi: 10.3389/fpsyt.2024.1414439.

Monosov, Ilya E., Jan Zimmermann, Michael J. Frank, Mackenzie W. Mathis, and Justin T. Baker. 2024. “Ethological Computational Psychiatry: Challenges and Opportunities.” *Current Opinion in Neurobiology* 86:102881. doi: 10.1016/j.conb.2024.102881.

Monteith, Scott, Tasha Glenn, John R. Geddes, Eric D. Achtyes, Peter C. Whybrow, and Michael Bauer. 2023. “Challenges and Ethical Considerations to Successfully Implement Artificial Intelligence in Clinical Medicine and Neuroscience: A Narrative Review.” *Pharmacopsychiatry* 56(6):209–13. doi: 10.1055/a-2142-9325.

Monteith, Scott, Tasha Glenn, John Geddes, Peter C. Whybrow, Eric Achtyes, and Michael Bauer. 2022. “Expectations for Artificial Intelligence (AI) in Psychiatry.” *Current Psychiatry Reports* 24(11):709–21. doi: 10.1007/s11920-022-01378-5.

Morley, Jessica, Caio C. V. Machado, Christopher Burr, Josh Cowls, Indra Joshi, Mariarosaria Taddeo, and Luciano Floridi. 2020. “The Ethics of AI in Health Care: A Mapping Review.” *Social Science & Medicine* 260:113172. doi: 10.1016/j.socscimed.2020.113172.

Murray, Graham K., Tian Lin, Jehannine Austin, John J. McGrath, Ian B. Hickie, and Naomi R. Wray. 2021. “Could Polygenic Risk Scores Be Useful in Psychiatry?: A Review.” *JAMA Psychiatry* 78(2):210–19. doi: 10.1001/jamapsychiatry.2020.3042.

Ortiz, Abigail, and Benoit H. Mulsant. 2024. “Beyond Step Count: Are We Ready to Use Digital Phenotyping to Make Actionable Individual Predictions in Psychiatry?” *J Med Internet Res* 26:e59826. doi: 10.2196/59826.

Ostojic, Dijana, Paris Alexandros Lalousis, Gary Donohoe, and Derek W. Morris. 2024. “The Challenges of Using Machine Learning Models in Psychiatric Research and Clinical Practice.” *European Neuropsychopharmacology* 88:53–65. doi: 10.1016/j.euroneuro.2024.08.005.

Oudin, Antoine, Redwan Maatoug, Alexis Bourla, Florian Ferreri, Olivier Bonnot, Bruno Millet, Félix Schoeller, Stéphane Mouchabac, and Vladimir Adrien. 2023. “Digital Phenotyping: Data-Driven Psychiatry to Redefine Mental Health.” *Journal of Medical Internet Research* 25(1):e44502. doi: 10.2196/44502.

Parziale, Andrea, and Deborah Mascalzoni. 2022. “Digital Biomarkers in Psychiatric Research: Data Protection Qualifications in a Complex Ecosystem.” *Frontiers in Psychiatry* 13.

Pavlopoulos, Adrianos, Theodoros Rachiotis, and Ilias Maglogiannis. 2024. “An Overview of Tools and Technologies for Anxiety and Depression Management Using AI.” *Applied Sciences* 14(19). doi: 10.3390/app14199068.

Rahul, Jagdeep, Diksha Sharma, Lakhan Dev Sharma, Umakanta Nanda, and Achintya Kumar Sarkar. 2024. “A Systematic Review of EEG Based Automated Schizophrenia Classification through Machine Learning and Deep Learning.” *Frontiers in Human Neuroscience* 18.

Rocheteau, Emma. 2023. “On the Role of Artificial Intelligence in Psychiatry.” *The British Journal of Psychiatry* 222(2):54–57. doi: 10.1192/bjp.2022.132.

Saheb, Tahereh, Tayebeh Saheb, and David O. Carpenter. 2021. “Mapping Research Strands of Ethics of Artificial Intelligence in Healthcare: A Bibliometric and Content Analysis.” *Computers in Biology and Medicine* 135:104660. doi: 10.1016/j.compbiomed.2021.104660.

Sahin, D., L. Kambeitz-Ilankovic, S. Wood, D. Dwyer, R. Upthegrove, R. Salokangas, S. Borgwardt, P. Brambilla, E. Meisenzahl, S. Ruhrmann, F. Schultze-Lutter, R. Lencer, A. Bertolino, C. Pantelis, N. Koutsouleris, J. Kambeitz, and PRONIA Study Grp. 2024. “Algorithmic Fairness in Precision Psychiatry: Analysis of Prediction Models in Individuals at Clinical High Risk for Psychosis.” *BRITISH JOURNAL OF PSYCHIATRY* 224(2):55–65. doi: 10.1192/bjp.2023.141.

Shen, Francis X., Benjamin C. Silverman, Patrick Monette, Sara Kimble, Scott L. Rauch, and Justin T. Baker. 2022. “An Ethics Checklist for Digital Health Research in Psychiatry: Viewpoint.” *J Med Internet Res* 24(2):e31146. doi: 10.2196/31146.

Singhal, Sorabh, Danielle L. Cooke, Ricardo I. Villareal, Joel J. Stoddard, Chen-Tan Lin, and Allison G. Dempsey. 2024. “Machine Learning for Mental Health: Applications, Challenges, and the Clinician’s Role.” *Current Psychiatry Reports*. doi: 10.1007/s11920-024-01561-w.

Skorburg, Joshua August, Kieran O’Doherty, and Phoebe Friesen. 2024. “Persons or Data Points? Ethics, Artificial Intelligence, and the Participatory Turn in Mental Health Research.” *American Psychologist* 79(1):137–49. doi: 10.1037/amp0001168.

Smith, William R., Paul S. Appelbaum, Matthew S. Lebowitz, Sinan Gülöksüz, Monica E. Calkins, Christian G. Kohler, Raquel E. Gur, and Ran Barzilay. 2023. “The Ethics of Risk Prediction for Psychosis and Suicide Attempt in Youth Mental Health.” *The Journal of Pediatrics* 263:113583. doi: 10.1016/j.jpeds.2023.113583.

Starke, Georg, Eva De Clercq, Stefan Borgwardt, and Bernice Simone Elger. 2021. “Computing Schizophrenia: Ethical Challenges for Machine Learning in Psychiatry.” *Psychological Medicine* 51(15):2515–21. doi: 10.1017/S0033291720001683.

Stein, Olivia A., and Audrey Prost. 2024. “Exploring the Societal Implications of Digital Mental Health Technologies: A Critical Review.” *SSM - Mental Health* 6:100373. doi: 10.1016/j.ssmmh.2024.100373.

Straw, Isabel. 2021. “Ethical Implications of Emotion Mining in Medicine.” *Health Policy and Technology* 10(1):191–95. doi: 10.1016/j.hlpt.2020.11.006.

Sultan, Mubashir, Christin Scholz, and Wouter van den Bos. 2023. “Leaving Traces behind: Using Social Media Digital Trace Data to Study Adolescent Wellbeing.” *Computers in Human Behavior Reports* 10:100281. doi: 10.1016/j.chbr.2023.100281.

Tabb, Kathryn, and Maël Lemoine. 2021. “The Prospects of Precision Psychiatry.” *Theoretical Medicine and Bioethics* 42(5):193–210. doi: 10.1007/s11017-022-09558-3.

Tejavibulya, Link, Max Rolison, Siyuan Gao, Qinghao Liang, Hannah Peterson, Javid Dadashkarimi, Michael C. Farruggia, C. Alice Hahn, Stephanie Noble, Sarah D. Lichenstein, Angeliki Pollatou, Alexander J. Dufford, and Dustin Scheinost. 2022. “Predicting the Future of Neuroimaging Predictive Models in Mental Health.” *Molecular Psychiatry* 27(8):3129–37. doi: 10.1038/s41380-022-01635-2.

Terra, Mohamed, Mohamed Baklola, Shaimaa Ali, and Karim El-Bastawisy. 2023. “Opportunities, Applications, Challenges and Ethical Implications of Artificial Intelligence in Psychiatry: A Narrative Review.” *The Egyptian Journal of Neurology, Psychiatry and Neurosurgery* 59(1):80. doi: 10.1186/s41983-023-00681-z.

Thieme, Anja, Danielle Belgrave, and Gavin Doherty. 2020. “Machine Learning in Mental Health: A Systematic Review of the HCI Literature to Support the Development of Effective and Implementable ML Systems.” *ACM Trans. Comput.-Hum. Interact.* 27(5):34:1-34:53. doi: 10.1145/3398069.

Torous, John, Sandra Bucci, Imogen H. Bell, Lars V. Kessing, Maria Faurholt-Jepsen, Pauline Whelan, Andre F. Carvalho, Matcheri Keshavan, Jake Linardon, and Joseph Firth. 2021. “The Growing Field of Digital Psychiatry: Current Evidence and the Future of Apps, Social Media, Chatbots, and Virtual Reality.” *World Psychiatry* 20(3):318–35. doi: 10.1002/wps.20883.

Upreti, Ramesh, Pedro G. Lind, Ahmed Elmokashfi, and Anis Yazidi. 2024. “Trustworthy Machine Learning in the Context of Security and Privacy.” *International Journal of Information Security* 23(3):2287–2314. doi: 10.1007/s10207-024-00813-3.

Vale, Mira D. 2024. “Moral Entrepreneurship and the Ethics of Artificial Intelligence in Digital Psychiatry.” *Socius* 10:23780231241259641. doi: 10.1177/23780231241259641.

Wang, Min, Zhoukang Wu, Xiaochu Zhang, Xiaosong He, and Liangjiecheng Huang. 2024. “Computing Addiction: Epistemic Injustice Challenges in the Culture of Computational Psychiatry.” *Acta Bioethica*. doi: 10.4067/s1726-569x2024000200263.

Wiese, Wanja, and Karl J. Friston. 2022. “AI Ethics in Computational Psychiatry: From the Neuroscience of Consciousness to the Ethics of Consciousness.” *Behavioural Brain Research* 420:113704. doi: 10.1016/j.bbr.2021.113704.

Wouters, Roel H. P., Marte Z. van der Horst, Cora M. Aalfs, Janita Bralten, Jurjen J. Luykx, and Janneke R. Zinkstok. 2024. “The Ethics of Polygenic Scores in Psychiatry: Minefield or Opportunity for Patient-Centered Psychiatry?” *Psychiatric Genetics* 34(2).

Wray, Naomi R., Tian Lin, Jehannine Austin, John J. McGrath, Ian B. Hickie, Graham K. Murray, and Peter M. Visscher. 2021. “From Basic Science to Clinical Application of Polygenic Risk Scores: A Primer.” *JAMA Psychiatry* 78(1):101–9. doi: 10.1001/jamapsychiatry.2020.3049.

Zhang, Melody, Jillian Scandiffio, Sarah Younus, Tharshini Jeyakumar, Inaara Karsan, Rebecca Charow, Mohammad Salhia, and David Wiljer. 2023. “The Adoption of AI in Mental Health Care–Perspectives From Mental Health Professionals: Qualitative Descriptive Study.” *JMIR Form Res* 7:e47847. doi: 10.2196/47847.

Zidaru, Teodor, Elizabeth M. Morrow, and Rich Stockley. 2021. “Ensuring Patient and Public Involvement in the Transition to AI-Assisted Mental Health Care: A Systematic Scoping Review and Agenda for Design Justice.” *Health Expectations* 24(4):1072–1124. doi: 10.1111/hex.13299.
