# Supplementary material for: Ethical decision-making for AI in mental health: the Integrated Ethical Approach for Computational Psychiatry (IEACP) framework
Source: Psychol Med. 2025 Jul 24;55:e213. doi: 10.1017/S0033291725101311 (PMC12315656; doi:10.1017/S0033291725101311)
Supplement: Putica et al. supplementary material [file S0033291725101311sup001.zip › Supplementaty Table 1_PM.docx]

**Supplementary Table S-1**

*Data Extraction Results from Literature Review on Ethical Considerations in Computational Psychiatry (N = 83)*

| Study | Study Type/ Focus | Ethical Challenges Identified | Ethical Values Identified/Addressed | Implementation Context |
| --- | --- | --- | --- | --- |
| (Ahmed and Hens 2022) | Review of microbiome big data and microbiome-based interventions in precision psychiatry | Privacy concerns due to microbiome's ability to identify individuals Unknown health risks of microbiome manipulation Impact of microbiome on personal identity and autonomy Stigmatization related to mental illness data | Autonomy/informed consent  Justice/equity  Privacy/confidentiality  Transparency/explainability | Use of microbiome-based interventions for mental health treatment Managing biobanks for microbiome big data Ethical frameworks for protecting sensitive data |
| (Arbanas 2024) | Review of ChatGPT and chatbot applications in psychiatry, focusing on clinical support and therapeutic use | Privacy and data security risks Ethical responsibility for AI errors Bias in AI outputs Lack of human oversight and empathy | Justice/equity  Beneficence/non-maleficence  Privacy/confidentiality  Transparency/explainability | Use of ChatGPT for clinical decision-making, therapy support, patient education, and crisis management Need for careful integration and ongoing oversight of AI tools in psychiatry |
| (Avula and Amalakanti 2024) | Updated review of AI applications, trends, and challenges in psychiatry | Privacy risks with AI-based data collection Algorithmic bias Opacity in black-box models Automation bias impacting clinical decision-making De-skilling of clinical staff | Beneficence/non-maleficence  Autonomy/informed consent  Justice/equity  Privacy/confidentiality  Transparency/explainability | Use of AI for diagnosis, monitoring, and intervention Integration of speech recognition, digital gaming, wearable devices, and machine learning in psychiatry |
| (Ball, Kalinowski, and Williams 2020) | Ethical analysis of implementing precision psychiatry approaches into clinical practice | Informed consent for novel precision psychiatry tools Risk of harm due to poor implementation Gaps in clinician competency for novel tools Inequitable distribution of precision psychiatry benefits | Beneficence/non-maleficence  Autonomy/informed consent  Justice/equity  Privacy/confidentiality  Transparency/explainability | Use of neuroimaging, genetics, and machine learning for treatment selection in psychiatry Addressing ethical safeguards for clinician training and equitable access to precision psychiatry |
| (Bentley et al. 2021) | Systematic review of safety and ethical practices for monitoring self-injurious thoughts and behaviors (SITBs) in real-time studies | Managing suicide risk in real-time monitoring Determining thresholds for intervention Balancing data accuracy with participant safety concerns Privacy and informed consent for sensitive data collection | Beneficence/non-maleficence  Autonomy/informed consent  Justice/equity  Privacy/confidentiality  Scientific integrity/validity | Use of ecological momentary assessment (EMA) and digital tools for SITB research Systematic review of monitoring protocols, including automated alerts and risk assessments |
| (Bertl, Ross, and Draheim 2022) | Systematic review of AI and decision support systems (DSS) in psychiatry | Privacy concerns with fragmented data systems Bias from poorly curated datasets Lack of transparency in AI outputs Limited generalizability and low maturity of tools | Autonomy/informed consent  Justice/equity  Privacy/confidentiality  Transparency/explainability  Scientific integrity/validity | AI tools for diagnosis, prediction, and treatment planning in psychiatry Identifies gaps in data quality, standardization, and real-world testing |
| (Briganti 2023) | Review of AI applications in psychiatry, focusing on diagnosis, treatment, and research directions | Privacy and confidentiality risks Bias and fairness issues in AI models Lack of transparency and explainability Ethical responsibility and accountability | Beneficence/non-maleficence  Autonomy/informed consent  Justice/equity  Privacy/confidentiality  Transparency/explainability | Application of AI techniques for diagnosis, hypothesis generation, and treatment predictions in psychiatry Emphasis on ethical frameworks and responsible use of AI |
| (Chekroud et al. 2021) | Review of machine learning applications for predicting treatment outcomes in psychiatry | Bias and fairness issues in training datasets Lack of transparency in machine learning models (black box problem) Responsibility for AI-driven decisions Potential for clinician disempowerment | Beneficence/non-maleficence Autonomy/informed consent  Justice/equity  Privacy/confidentiality  Transparency/explainability | Use of machine learning to predict treatment response for medications, psychotherapy, and neurobiological interventions Integration of electronic health records, smartphone data, and neuroimaging into predictive models |
| (I. Chen et al. 2022) | Review of mobile apps for the development of digital biomarkers for personalized diagnosis and treatment of mental illnesses | Privacy risks from continuous data collection Ethical use of passive data tracking Potential misuse of sensitive personal health data | Beneficence/non-maleficence  Autonomy/informed consent  Justice/equity  Privacy/confidentiality  Transparency/explainability | Use of mobile apps to monitor human–smartphone interactions for mental health research Applications in circadian rhythm tracking, work hours monitoring, and mental illness prediction |
| (Z. S. Chen et al. 2022) | Review of machine learning (ML) applications in precision psychiatry | Privacy and data security risks Bias in datasets and models Transparency of black-box models Over-reliance on ML predictions | Beneficence/non-maleficence  Autonomy/informed consent  Justice/equity  Privacy/confidentiality  Transparency/explainability | Use of ML and AI in neuroimaging, digital phenotyping, and wearable devices for diagnosis and treatment prediction Integration challenges across multimodal data sources |
| (Clarke, Foltz, and Garrard 2020) | A review exploring Natural Language Processing and machine learning approaches for detecting early signs of Alzheimer's disease through language analysis | Issues with collection and storage of patient discourse data for safety/privacy protection Problems with openly shared data having unreliable diagnoses Constraints around obtaining consent for data re-use  Privacy concerns with personal histories  Diagnostic uncertainty (only confirmed post-mortem)  Clinical acceptance issues around AI interpretability vs performance  Trust and accountability concerns with automated systems  Data security with remote monitoring  Transparency around data collection/usage  Questions about ease of use/intrusiveness  Concerns about accountability for errors  Questions about benefit of early detection given lack of treatment | Beneficence/non-maleficence  Autonomy/informed consent Justice/equity Privacy/confidentiality Transparency/explainability Scientific integrity/validity | Research setting: large-scale data collection/analysis, brain banking correlation, diagnostic tool development  Clinical setting: early detection tools, disease monitoring, treatment assessment  Real-world considerations: stakeholder involvement needed (researchers, clinicians, patients, health services, commercial enterprises), clear accountability frameworks required, trust-building measures important, need for ongoing effectiveness evaluation |
| (D’Alfonso 2020) | Review on AI applications in mental health, focusing on digital phenotyping, language analysis, and chatbots | Privacy risks from digital phenotyping and data mining Ethical implications of AI-based chatbots in mental health Bias in natural language processing and model training | Autonomy/informed consent  Justice/equity Privacy/confidentiality  Transparency/explainability  Scientific integrity/validity | Use of smartphone sensing and digital exhaust for mental health prediction AI-based language analysis for diagnosis and detection of mental illness Application of AI-driven chatbots for therapeutic interventions |
| (D’Souza et al. 2024) | Mixed methods study evaluating psychiatrists' perspectives and ethical understanding of AI implementation through workshop | Limited knowledge and exposure to AI among psychiatrists  Concerns about patient privacy and data protection  Challenges with informed consent  Impact on doctor-patient relationship  Risks of AI making clinical decisions Potential dehumanization of care  Accessibility and usability concerns  Risk of clinical workflow disruption | Beneficence/non-maleficence  Autonomy/informed consent  Justice/equity  Privacy/confidentiality  Transparency/explainability  Scientific integrity/validity | Educational workshop context  Clinical practice integration  Need for ongoing training programs  Requirement for regulatory frameworks  Need for collaborative development  Focus on ethical guideline development |
| (Davidson 2022) | Critical analysis of digital phenotyping in psychiatry, focusing on its promises, methodological issues, and ethical concerns | Privacy and security risks with passive digital trace data Lack of transparency in data collection and analysis Ethical use of invasive monitoring for vulnerable populations Data misuse and bias | Autonomy/informed consent Justice/equity  Privacy/confidentiality  Transparency/explainability  Scientific integrity/validity | Use of smartphone-based data, wearables, and environmental sensors for mental health monitoring Integration challenges into healthcare systems and ethical regulation gaps |
| (Diaz-Asper et al. 2024) | Framework for language technologies in behavioral research and clinical applications focusing on ethical issues | Privacy concerns from speech data collection Black box problem (lack of explainability) Algorithmic bias Human agency and oversight gaps | Autonomy/informed consent Justice/equity Privacy/confidentiality  Transparency/explainability | Development and application of NLP and AI systems in psychological and clinical research Addressing ethical gaps in APA guidelines |
| (Dikaios et al. 2023) | Review of speech analysis applications in psychiatry for diagnosis, severity assessment, and prediction | Privacy concerns from speech data collection Potential for bias in AI/ML models Risks of dehumanization in automated assessment | Justice/equity  Privacy/confidentiality  Transparency/explainability  Scientific integrity/validity | Use of speech features for diagnosis, severity monitoring, and treatment prediction in psychiatry Application of AI/ML in clinical psychiatry workflows |
| (Dube et al. n.d.) | Ethical analysis of artificial intelligence (AI) use in psychiatry, with a focus on vulnerable populations | Privacy concerns with sensitive data collection Algorithmic bias exacerbating health inequities Lack of transparency and explainability in AI decisions Disparities in access to AI technologies  Ethical responsibility for errors | Justice/equity Privacy/confidentiality  Transparency/explainability  Scientific integrity/validity | Applications of AI in child and adolescent psychiatry Integration of AI for diagnosis, treatment planning, and reducing clinician burden Global inequities in AI deployment due to resource gaps |
| (Dwyer and Koutsouleris 2022) | Review of machine learning applications in child and adolescent psychiatry, focusing on diagnosis, prognosis, and treatment prediction | Data privacy and security issues with sensitive child and adolescent information Algorithmic bias affecting diverse populations Generalizability concerns of predictive models across contexts Ethical use of machine learning in early intervention | Beneficence/non-maleficence  Justice/equity Privacy/confidentiality  Transparency/explainability  Scientific integrity/validity | Use of ML for early detection and treatment selection in autism, ADHD, depression, and psychosis Importance of validating models for clinical translation and real-world use |
| (Espejo, Reiner, and Wenzinger 2023) | Review of AI's role in mental healthcare, exploring its benefits, limitations, and future directions | Privacy concerns with sensitive health data Algorithmic bias and lack of self-reflection in AI Overreliance on AI tools replacing human care Risk of entrenching disparities in access | Justice/equity  Privacy/confidentiality  Transparency/explainability  Scientific integrity/validity | Use of AI in diagnostics, real-time patient monitoring, neuroimaging, and psychotherapy applications Emphasis on clinician involvement in AI regulation and ethical oversight |
| (Faissner et al. 2024) | Ethical analysis of epistemic injustice in passive self-tracking apps for depression detection | Hermeneutical gaps in understanding mental illness Algorithm opacity and black box issues  Bias towards biostatistical understanding of depression  Risk of testimonial injustice in healthcare encounters Marginalization of alternative interpretations of mental health Risk of contributing to existing power imbalances | Autonomy/informed consent  Justice/equity  Scientific integrity/validity | Direct-to-consumer use  Clinical healthcare encounters Mental healthcare settings Need for participatory development approaches Need for integration of diverse epistemic resources Need for combining passive tracking with user interaction |
| (Fardouly, Crosby, and Sukunesan 2022) | Narrative review on machine learning (ML) for the detection, prevention, and treatment of eating disorders | Privacy concerns with data collection Algorithmic bias affecting diverse populations Ethical use of social media and ML models for early intervention Misuse of digital data and stigmatization risks | Beneficence/non-maleficence  Justice/equity  Privacy/confidentiality  Transparency/explainability | Use of machine learning for detecting eating disorder risk through social media posts, surveys, and neuroimaging Focus on scalable prevention programs and real-time interventions |
| (Farmer et al. 2024) | Review of ethical and practical considerations for AI integration in psychological practice | Privacy and confidentiality risks with sensitive data Algorithmic bias and perpetuation of inequity Lack of transparency and explainability in AI outputs Deskilling and automation bias for clinicians | Autonomy/informed consent Justice/equity Privacy/confidentiality  Transparency/explainability  Scientific integrity/validity | Use of AI tools for report generation, decision-making support, and administrative burden reduction Integration of AI-driven chatbots for therapy and psychoeducation Guidelines for ethical implementation and clinician oversight |
| (Fisher 2024) | Ethical analysis of real-world AI tools in clinical psychiatry | Privacy and data security risks Algorithmic bias impacting care access and outcomes Seductive allure and over-reliance on AI tools Loss of clinician reflection and skill development Liability for AI-driven decisions | Justice/equity  Privacy/confidentiality  Transparency/explainability  Scientific integrity/validity | Applications of AI in screening, intake, documentation, decision support, and adjunctive treatments Ethical challenges arising from overreliance and misuse of AI tools in psychiatry |
| (Fusar-Poli et al. 2022) | Critical review and framework development for precision psychiatry | Clinical prediction models may not be accurate enough to capture complexity of mental disorders  Poor explainability of complex AI models ("black-box")  Generalizability and bias concerns for vulnerable populations Privacy concerns with sensitive psychiatric data  Risk of over-reliance on computational tools Communication challenges regarding risk estimates  Data leakage and privacy concerns  Potential to exacerbate healthcare disparities  Methods of disclosure poorly investigated | Beneficence/non-maleficence  Autonomy/informed consent  Justice/equity  Privacy/confidentiality  Transparency/explainability  Scientific integrity/validity | Need for structured implementation frameworks  Integration into clinical workflows  Training requirements for healthcare providers  Need for cost-effectiveness research  Requirements for data governance and security  Need for continuous monitoring and validation  Focus on vulnerable populations  Need for mental health literacy development |
| (Galderisi et al. 2024) | Ethical overview of challenges in contemporary psychiatry, including human rights, digital psychiatry, and coercion | Human rights violations in mental health care Digital psychiatry privacy issues and AI bias Risk of overmedicalization in early interventions Ethical issues in end-of-life decisions Conflict of interest in clinical research | Beneficence/non-maleficence  Autonomy/informed consent  Justice/equity  Privacy/confidentiality | Application of human rights frameworks to psychiatry Ethical integration of digital tools and AI in clinical practice Addressing coercive practices with alternatives Early intervention ethics and shared decision-making processes |
| (Gillett and Saunders 2019) | Review of remote monitoring in psychiatry focusing on mood and anxiety disorders | Privacy and user autonomy concerns Coercion risk in long-term monitoring Risk of medicalizing everyday experiences Data security and storage risks Stigma and discrimination concerns | Beneficence/non-maleficence  Autonomy/informed consent Privacy/confidentiality  Transparency/explainability  Scientific integrity/validity | Clinical assessment settings Research environments Integration with traditional clinical methods Need for validation strategies Requirement for clinician oversight |
| (Gooding and Kariotis 2021) | Scoping review of ethical and legal challenges for algorithmic and data-driven technologies in mental health care | Privacy risks and inadequate data protection  Algorithmic bias and fairness concerns Lack of transparency in AI systems Minimal involvement of service users in AI development Overmedicalization and techno-solutionism | Autonomy/informed consent  Justice/equity  Privacy/confidentiality  Transparency/explainability  Scientific integrity/validity | Application of AI and digital technologies in diagnosis, public health monitoring, and treatment prediction Highlights the lack of explicit ethical considerations in many applied studies and the need for participatory development |
| (Graham et al. 2019) | Overview of AI applications in mental health and ethical implications for clinical practice | Privacy and data security Potential for bias in AI models Over-reliance on AI and loss of clinical judgment Transparency and explainability | Beneficence/non-maleficence  Autonomy/informed consent  Privacy/confidentiality  Transparency/explainability | Use of AI in electronic health records (EHR), brain imaging, and digital tools for predicting and diagnosing mental illnesses Emphasis on integrating AI into clinical workflows |
| (Gültekin and Şahin 2024) | Qualitative study on Turkish mental health professionals’ perspectives on AI in mental health services | Privacy and data security concerns Algorithmic bias The "black box" problem and lack of transparency Ethical and legal uncertainties regarding accountability | Beneficence/non-maleficence  Justice/equity  Privacy/confidentiality  Transparency/explainability  Scientific integrity/validity | Applications of AI in psychotherapy, diagnostics, and mental health interventions Professionals view AI as an assistant rather than a replacement for clinicians |
| (Hagendorff 2021) | Conceptual paper proposing an ethical data filtering approach to improve machine learning models | Privacy concerns with extensive data tracking Algorithmic bias caused by poor training data quality Ethical selection of "morally sound" data sources | Beneficence/non-maleficence  Justice/equity  Privacy/confidentiality  Transparency/explainability  Scientific integrity/validity | Development of supervised machine learning using ethically pre-filtered data for tasks like autonomous driving, search engines, and recommendation systems |
| (Hart et al. 2022) | Empirical study using smartphone sensors and machine learning for ecological momentary assessment | Battery drain and device responsiveness Personal identifiability of data Privacy of sensor data collection Informed consent for complex data collection Balance between data collection and privacy | Privacy/confidentiality  Scientific integrity/validity | Real-world data collection during questionnaire completion Need for standardized data collection protocols Consideration of device capabilities |
| (Heinrichs and Eickhoff 2020) | Conceptual discussion on the ethical issues of machine learning algorithms for medical diagnosis and prediction | Epistemic opacity (black-box problem) undermines understanding and trust Challenges in assigning responsibility for AI-based failures Lack of explainability in AI systems | Autonomy/informed consent  Justice/equity  Scientific integrity/validity | Use of machine learning for medical diagnosis and prediction in psychiatry and neuroimaging Advocates for explainable AI to improve interpretability and responsibility in clinical applications |
| (Hurley et al. 2024) | Multi-site qualitative study examining ethical considerations in computer perception and neurotechnology integration | Invasiveness of passive/continuous data collection  Data protection and security risks  Limited vs. hyper awareness of monitoring Integration challenges with neural data  Privacy in non-clinical settings | Beneficence/non-maleficence  Autonomy/informed consent  Justice/equity  Privacy/confidentiality  Transparency/explainability | Clinical integration of computer perception technologies  Need for human supervision  Training requirements  Data protection protocols  Regulatory compliance needs |
| ​(Islam et al. 2024) | Review of machine learning models and methods for detecting and predicting mental health conditions | Bias in data and algorithms Limited generalizability Privacy and ethical considerations | Justice/equity  Privacy/confidentiality  Scientific integrity/validity | Use of heterogeneous datasets Focus on integrating social media, wearable sensors, and audio data analysis  Personalized support systems |
| (Jacobson et al. 2020) | Ethical dilemmas in mobile health and machine learning applications in psychiatry research | Privacy concerns with passive data collection Challenges in obtaining informed consent for digital tools Monitoring and intervening during adverse events Algorithmic bias and fairness issues Lack of transparency in machine learning models | Beneficence/non-maleficence  Autonomy/informed consent  Justice/equity  Privacy/confidentiality  Transparency/explainability | Application of machine learning to analyse mobile health data for psychiatric monitoring and prediction Ethical obligations for researchers to balance privacy and scientific progress |
| (Jin et al. 2023) | Overview of AI applications in mental healthcare, focusing on diagnosis, treatment, and digital psychiatry | Privacy risks from AI-driven data collection Algorithmic bias and fairness issues Transparency of black-box models Overreliance on AI decision-making | Autonomy/informed consent  Justice/equity  Privacy/confidentiality  Transparency/explainability  Scientific integrity/validity | Applications of AI for diagnosis, treatment selection, and digital psychiatry tools like NLP, wearables, and chatbots Highlights the potential for AI to improve diagnosis and care delivery, while addressing ethical risks |
| (Kappen, Vanderhasselt, and Slavich 2023) | Review on speech as a biosignal for precision psychiatry to detect mental and physical health conditions | Privacy risks from continuous speech data collection Ethical concerns regarding consent for passive monitoring Data security vulnerabilities  Stigma and potential misuse of speech data | Beneficence/non-maleficence  Autonomy/informed consent  Justice/equity Privacy/confidentiality  Transparency/explainability | Use of speech as a biosignal to detect and predict stress, depression, schizophrenia, and other health conditions Focus on developing tailored just-in-time adaptive interventions (JITAIs) for clinical implementation |
| (Kirtley et al. 2022) | Focused review of machine learning applications in suicide research and prevention using EHR data | Data quality and bias  Transparency Privacy of health records Algorithmic bias Responsibility for intervention Communication of risk predictions | Beneficence/non-maleficence Autonomy/informed consent  Justice/equity  Privacy/confidentiality  Transparency/explainability  Scientific integrity/validity | Healthcare system integration  Clinical decision support Need for independent validation Training needs for clinicians Scalability of interventions Global health contexts |
| (Kleine et al. 2023) | Review of patents on AI-enabled precision psychiatry tools, focusing on diagnostic, prognostic, and treatment prediction models | Privacy concerns in the use of sensitive data Potential for algorithmic bias Lack of transparency in predictions Uneven access to AI-based tools | Justice/equity  Privacy/confidentiality  Transparency/explainability  Scientific integrity/validity | Use of behavioral, neuroimaging, and biological data sources for precision psychiatry Adoption of AI in clinical workflows Commercialization trends and regulation gaps |
| (Kolding et al. 2024) | Systematic review of generative AI applications in psychiatry and mental health care | Privacy risks with generative AI tools Ethical and safety concerns around unpredictable outputs Algorithmic bias and lack of transparency in generative models Limited clinical relevance of AI tools in their current state | Beneficence/non-maleficence Justice/equity  Privacy/confidentiality  Transparency/explainability  Scientific integrity/validity | Applications of generative AI, particularly ChatGPT, in psychiatry for education, diagnosis, and psychoeducation Emphasis on improving transparency, user involvement, and clinical trials for ethical implementation |
| (Koutsouleris et al. 2022) | Review of AI applications in precision psychiatry and mental health care implementation challenges | Privacy risks with AI-driven data collection Bias and fairness in AI models, particularly for marginalized groups  Overreliance on AI leading to loss of clinician reflection and autonomy Transparency and explainability of AI algorithms Ethical safeguards for AI errors and unintended harm | Beneficence/non-maleficence Justice/equity Privacy/confidentiality  Transparency/explainability  Scientific integrity/validity | Application of AI tools for diagnosis, prediction, and therapeutic decision-making in mental health care Emphasis on integrating AI into clinical workflows while safeguarding ethical standards and patient trust |
| (Leung 2023) | Conceptual paper on using AI and machine learning to augment social media for telehealth and patient monitoring | Privacy risks with AI–ML-based social media tools  Spread of misinformation on social media platforms Ethical use of user data for research purposes Transparency in algorithmic decision-making | Beneficence/non-maleficence  Justice/equity  Privacy/confidentiality  Transparency/explainability  Scientific integrity/validity | AI–ML applications for remote patient monitoring, telehealth marketing, and public health education Development of privacy-enhancing technologies and safeguards to address privacy issues |
| (Levkovich, Shinan-Altman, and Elyoseph 2024) | Experimental study examining cultural sensitivity of ChatGPT-3.5 and ChatGPT-4 in suicide risk assessment using vignette methodology | Cultural biases and racism in AI assessment | Beneficence/non-maleficence  Justice/equity  Privacy/confidentiality  Transparency/explainability  Scientific integrity/validity | Mental health clinical settings  Suicide risk assessment protocols  Cultural validation requirements  Clinician oversight needs  Training data considerations  Clinical workflow integration  Supervision protocols  Quality assurance systems |
| (Lewis et al. 2024) | Case study on ethical challenges in managing differential performance of polygenic risk scores (PRSs) | Health disparities due to differential PRS performance across populations Ethical implications of group-based PRS validation Risk of conflating genetic ancestry with race and ethnicity Challenges in communicating PRS limitations to diverse patient groups | Autonomy/informed consent  Justice/equity  Transparency/explainability  Scientific integrity/validity | Application of PRSs for 10 chronic conditions within the eMERGE network Implementation framework for diverse and historically underserved populations Ethical communication strategies developed to address group-based performance concerns |
| (Manchia et al. 2020) | Narrative review on precision medicine in psychiatry, focusing on clinical, neuroimaging, and biological data | Privacy concerns due to the analysis of massive datasets Self-determination and informed consent Equity in access to precision tools Stigmatization risks | Beneficence/non-maleficence  Autonomy/informed consent  Justice/equity  Privacy/confidentiality | Use of machine learning to predict treatment response and outcomes Integration of biological, clinical, and neuroimaging data in precision psychiatry |
| (Mazor et al. 2023) | Conceptual analysis on the ethical role of consciousness research and its implications for moral consideration | Ethical implications of consciousness research influencing moral status of animals and humans Use of invasive experiments on animals assumed to be conscious Conflict between scientific validity and moral justification Risks of cultural and societal biases shaping the attribution of consciousness | Beneficence/non-maleficence  Justice/equity  Transparency/explainability  Scientific integrity/validity | Focus on neuroscientific research in humans, animals, and clinical populations to define and measure consciousness Emphasizes the need for conceptual clarity and ethical reflection in studies of animal and human consciousness |
| (McCradden, Hui, and Buchman 2023) | Conceptual analysis of the ethical challenges of integrating AI in psychiatric clinical decision-making | Epistemic privileging of AI systems over clinician and patient knowledge Algorithmic bias and opacity (black-box problem) Risk of epistemic injustice, particularly testimonial and hermeneutical injustice Misuse of AI outputs for decisions about treatment and care pathways | Autonomy/informed consent  Justice/equity  Transparency/explainability Scientific integrity/validity | Use of AI systems for psychiatric prediction, diagnosis, and treatment recommendations Advocates for balancing AI outputs with clinical expertise and patient experiences to avoid harms like over-reliance and bias |
| (Monaco et al. 2024) | Study protocol for developing an AI platform for personalized treatment of eating disorders | Privacy and data security risks Ethical implications of AI-driven interventions and real-time data collection Bias in AI predictions due to incomplete data Transparency and explainability concerns | Autonomy/informed consent  Justice/equity  Privacy/confidentiality  Transparency/explainability  Scientific integrity/validity | AI-based data integration for personalized treatment plans, relapse prediction, and therapy optimization for eating disorders Implementation of a chatbot for patient engagement and support |
| (Monosov et al. 2024) | Review on opportunities and challenges in computational psychiatry for long-term behavioral studies | Privacy concerns from continuous behavioral monitoring Bias in AI models and neural data interpretation Ethical use of AI in mental health interventions | Autonomy/informed consent  Justice/equity  Privacy/confidentiality  Transparency/explainability  Scientific integrity/validity | Integration of AI, machine learning, and wearable sensors to monitor mood, cognition, and behavior over extended timescales Bridging animal and human data for personalized treatment strategies |
| (Monteith et al. 2023) | Narrative review exploring barriers to AI maturity and adoption in psychiatry and clinical medicine | Data quality issues (e.g., missing data, bias in EHR) Lack of transparency in AI systems (black-box problem) Dataset shift impacting performance Human factors: automation bias, deskilling, and clinician trust concerns Safety risks due to unanticipated AI failures | Beneficence/non-maleficence Justice/equity  Transparency/explainability  Scientific integrity/validity | Discussion on challenges of integrating AI into psychiatry for clinical decision-making, diagnostics, and workflow optimization Emphasis on building AI maturity with appropriate validation, regulation, and clinician education |
| (Monteith et al. 2023) | Narrative review on ethical challenges of AI prediction tools in psychiatry and clinical neuroscience | Data quality issues in electronic medical records (missing data, bias) Transparency and explainability in AI systems (black-box problem) Dataset shift leading to poor model generalizability Overreliance on AI leading to deskilling and automation bias | Beneficence/non-maleficence  Autonomy/informed consent  Privacy/confidentiality  Transparency/explainability  Scientific integrity/validity | Implementation of AI tools for predicting treatment outcomes and improving clinical workflows Emphasis on the need for human oversight, error tracking, and ethical deployment in psychiatric care |
| (Morley et al. 2020) | Mapping review of ethical risks related to AI implementation in healthcare | Epistemic risks: inconclusive, inscrutable, and misguided evidence Normative risks: unfair outcomes, bias, and transformative effects Traceability issues: difficulty assigning responsibility and liability in AI-based systems | Justice/equity  Transparency/explainability  Scientific integrity/validity | Application of AI for clinical decision support, diagnostics, and health system optimization Framework categorizing ethical risks across six levels of abstraction: individual, interpersonal, group, institutional, sectoral, and societal |
| (Murray et al. 2021) | Clinical review evaluating potential utility of polygenic risk scores (PRS) in psychiatry | Privacy and security of genetic data  Risk of discrimination Issues around return of results  Challenges in patient understanding, Risk of deterministic interpretations Equity concerns around ancestry differences in PRS accuracy Challenges in clinical implementation | Beneficence/non-maleficence  Autonomy/informed consent Justice/equity  Privacy/confidentiality  Transparency/explainability  Scientific integrity/validity | Clinical psychiatry settings Research context developing evidence base Focus on practical implementation considerations  Youth mental health services  General psychiatric practice |
| (Ortiz and Mulsant 2024) | Viewpoint on the challenges and opportunities of digital phenotyping to make actionable predictions in psychiatry | Privacy concerns from intense monitoring of behavior and mental states Lack of explainability ("black box" problem) Implementation gaps in clinical practice | Autonomy/informed consent  Privacy/confidentiality  Transparency/explainability  Scientific integrity/validity | Digital phenotyping for individualized, timely treatment Bridging gaps in predictive models for clinical psychiatry outcomes |
| (Ostojic et al. 2024) | Review of challenges in using machine learning (ML) models in psychiatric research and clinical practice | Data quality and "curse of dimensionality" with high-dimensional datasets Algorithmic bias and lack of external validation The "black-box" problem and lack of transparency in ML models Missing data and data leakage concerns | Beneficence/non-maleficence  Transparency/explainability  Scientific integrity/validity | Application of ML models to improve psychiatric diagnosis, prognosis, and treatment selection Focus on handling challenges like data imbalance, explainability, and validation for clinical adoption |
| (Oudin et al. 2023) | Viewpoint on digital phenotyping and its role in redefining mental health | Privacy and confidentiality risks Algorithmic bias and ethical design of digital tools Dehumanization of care Normativity risks (arbitrary standards) Black-box opacity and over-reliance on data | Autonomy/informed consent  Justice/equity  Privacy/confidentiality  Transparency/explainability | Use of digital phenotyping for continuous patient monitoring, diagnosis, and therapy optimization Focus on balancing technological integration with preserving human-centered care and agency in psychiatry |
| (Parziale and Mascalzoni 2022) | Analysis of data protection and privacy issues in the use of digital biomarkers in psychiatric research | Privacy and data protection concerns Trust and transparency Risks of stigma and discrimination Data governance gaps | Privacy/confidentiality  Transparency/explainability  Scientific integrity/validity | Compliance with GDPR in digital biomarker-powered research Balancing data sharing for psychiatric research while ensuring participant rights and trust |
| (Pavlopoulos, Rachiotis, and Maglogiannis 2024) | Review of AI tools and technologies for managing anxiety and depression | Privacy concerns with personal health data Overreliance on AI for mental health care Algorithmic bias in NLP tools | Autonomy/informed consent  Justice/equity  Privacy/confidentiality  Transparency/explainability | Application of chatbots, mobile apps, and LLMs for mental health care Addressing gaps in accessibility and personalization of treatments |
| (Rahul et al. 2024) | Systematic review on EEG-based schizophrenia classification using machine learning and deep learning | Privacy concerns regarding EEG data collection Algorithmic bias in ML/DL models Lack of transparency in deep learning models Data accessibility and reproducibility challenges | Justice/equity  Privacy/confidentiality  Transparency/explainability  Scientific integrity/validity | Application of ML and DL models to classify schizophrenia using EEG signals Emphasis on integrating EEG data with AI while addressing ethical challenges like data privacy and fairness |
| (Rocheteau 2023) | Analysis of AI roles in psychiatric diagnosis, monitoring, and treatment | Lack of intrinsic morality in AI systems Questions of responsibility in AI decisions AI interpretability and transparency Capacity and consent issues Privacy concerns with sensitive mental health data Bias and structural injustices in mental health data | Beneficence/non-maleficence  Autonomy/informed consent  Justice/equity  Privacy/confidentiality  Transparency/explainability  Scientific integrity/validity | Clinical diagnosis tools  Patient monitoring systems Treatment recommendation systems Need for human oversight Mental Health Act assessment contexts Integration with psychotherapy |
| (Saheb, Saheb, and Carpenter 2021) | Bibliometric and content analysis of research on AI ethics in healthcare | Data ethics and privacy concerns  Algorithm bias and transparency Safety of AI interventions  Clinical competency and education needs Human-machine interaction concerns  Robot ethics challenges  Patient autonomy and consent issues  Discriminatory consequences  Accountability and liability  Resource inequality  Surveillance concerns  Cultural adaptability challenges | Beneficence/non-maleficence  Autonomy/informed consent  Justice/equity  Privacy/confidentiality  Scientific integrity/validity | Clinical decision support systems  Mental healthcare delivery  Surgical and robotic interventions  Medical imaging analysis  Personalized medicine  Global healthcare systems  Chronic disease management  Clinical education and training  Research and development contexts Elderly care settings  ICU and emergency care  Public health surveillance |
| (Sahin et al. 2024) | Empirical analysis of algorithmic fairness in precision psychiatry prediction models for psychosis outcomes | Bias in AI models regarding education and gender Unequal predictive accuracy and false positives/negatives Lack of fairness validation in clinical AI use | Justice/equity  Transparency/explainability | Evaluation of AI predictions for psychosis transition and functional outcomes Comparison to clinician predictions as benchmarks |
| (Shen et al. 2022) | Viewpoint presenting an Ethics Checklist for digital health research in psychiatry, focusing on deep phenotyping | Privacy concerns from extensive data collection Lack of informed consent for complex passive data gathering Equity and diversity issues Legal gaps in regulatory frameworks | Beneficence/non-maleficence  Autonomy/informed consent  Justice/equity  Privacy/confidentiality  Transparency/explainability | Application of deep phenotyping approaches in psychiatry Development of the "Ethics Checklist" to guide ethical, legal, and social considerations in digital psychiatry research |
| (Singhal et al. 2024) | Review on machine learning (ML) applications, challenges, and the clinician's role in mental health care | Privacy and data security risks  Algorithmic bias and inequity Lack of transparency in ML models Automation bias and deskilling of clinicians Health equity concerns related to the digital divide | Beneficence/non-maleficence  Autonomy/informed consent  Justice/equity  Privacy/confidentiality  Transparency/explainability | Integration of ML in EHR for diagnosis, crisis prediction, and treatment response Use of mobile applications and remote monitoring tools for continuous patient care Emphasis on clinician involvement for ethical deployment |
| (Skorburg, O’Doherty, and Friesen 2024) | Conceptual paper on ethical tensions between AI/ML and participatory mental health research | Risk of erasing participant voices Over-reliance on proxies for lived experience Lack of patient inclusion in research governance Ethical issues in EHR data use | Beneficence/non-maleficence  Autonomy/informed consent  Justice/equity  Transparency/explainability  Scientific integrity/validity | Need for participatory research methods Integration of service-user governance Algorithmic impact assessments (AIAs) Public deliberation for policy guidance |
| (Smith et al. 2023) | Narrative literature review and ethical analysis of PRS and ERS use in youth mental health, focusing on psychosis and suicide risk prediction | Privacy/data security Patient autonomy vs. parental authority Algorithmic bias Early intervention benefits vs. false positive risks Consent in minors Direct-to-consumer testing "Black box" algorithm interpretability Risk communication Data sharing between stakeholders | Beneficence/non-maleficence  Autonomy/informed consent  Justice/equity  Privacy/confidentiality  Transparency/explainability  Scientific integrity/validity | Youth mental health settings Clinical high-risk programs Emergency departments Primary care Inpatient units Outpatient clinics |
| (Starke, De Clercq, Borgwardt, et al. 2021) | Ethical analysis of machine learning (ML) applications in psychiatry with a focus on schizophrenia | Privacy concerns Lack of transparency in ML algorithms ("black box problem") Risk of bias in training data  Diagnostic overshadowing | Beneficence/non-maleficence  Autonomy/informed consent  Justice/equity | Research on subtyping schizophrenia through neuroimaging data ML-based diagnostics and treatment recommendations |
| (Starke, De Clercq, and Elger 2021) | Theoretical/conceptual paper on addressing algorithmic bias in medical machine learning | Algorithmic bias Data fairness Difficulty distinguishing justified vs unjustified biases Lack of transparency in ML models Ethical evaluation of ML utility and fairness | Beneficence/non-maleficence  Autonomy/informed consent  Justice/equity  Transparency/explainability  Scientific integrity/validity | Clinical utility as a key evaluation measure Testing fairness across vulnerable populations Integration into regulatory processes Context-specific and disease-specific evaluations |
| (Stein and Prost 2024) | Critical review of societal implications of digital mental health technologies | Privacy breaches Dehumanization of care Overmedicalization Surveillance concerns Algorithmic bias | Autonomy/informed consent  Justice/equity  Privacy/confidentiality  Transparency/explainability  Scientific integrity/validity | Potential effects on healthcare access and quality Use of monitoring for clinical/public health systems Call for comprehensive regulation |
| (Straw 2021) | Review on the ethical implications of emotion mining in mental health using AI and digital phenotyping | Privacy breaches from emotion mining and digital phenotyping Algorithmic bias and fairness issues Lack of informed consent for inferred emotional states Risks of stigma and discrimination | Beneficence/non-maleficence  Autonomy/informed consent  Justice/equity  Privacy/confidentiality  Transparency/explainability  Scientific integrity/validity | Use of emotion mining in predicting self-harm, suicide, and public health surveillance Integrating AI tools into mental health systems while ensuring equity and ethical accountability |
| (Sultan, Scholz, and van den Bos 2023) | Review of social media digital trace data to study adolescent wellbeing | Privacy risks from digital trace data Ethical issues in informed consent for minors Potential for bias in big data analysis | Beneficence/non-maleficence  Autonomy/informed consent Justice/equity  Privacy/confidentiality  Transparency/explainability  Scientific integrity/validity | Use of social media trace data to explore adolescent mental health Proposals for multi-level digital phenotype frameworks |
| (Tabb and Lemoine 2021) | Review/Analysis examining precision medicine paradigm in psychiatry | Challenges of adapting precision medicine framework from oncology to psychiatry  Risk of over-emphasis on biomarkers without sufficient evidence Risk of abandoning useful diagnostic categories prematurely  Challenges in achieving reliable localization of psychiatric disorders  Risk of technological solutionism | Scientific integrity/validity | Academic/theoretical context examining foundations and justification of precision psychiatry  Focus on conceptual and epistemological challenges of precision approach in psychiatry |
| (Tejavibulya et al. 2022) | Review on the future of neuroimaging-based predictive models in mental health research | Bias and fairness in machine learning models  Dirty data (missing, noisy, or biased data) Generalizability issues across populations | Beneficence/non-maleficence  Autonomy/informed consent  Justice/equity  Transparency/explainability  Scientific integrity/validity | Application of neuroimaging-based models for predictive psychiatry Addressing data bias and improving model generalizability |
| (Terra et al. 2023) | Narrative review on AI opportunities, applications, and ethical implications in psychiatry | Privacy and data security risks Algorithmic bias and unequal treatment  Transparency and accountability concerns Ethical risks of automation impacting therapeutic relationships Over-reliance on AI and patient autonomy | Autonomy/informed consent  Justice/equity  Privacy/confidentiality  Transparency/explainability  Scientific integrity/validity | Applications in mental health monitoring, diagnosis, prediction, and treatment using AI and deep learning Emphasis on ethical AI integration to mitigate risks of bias and ensure responsible, human-centered AI use |
| (Thieme, Belgrave, and Doherty 2020) | Systematic review of machine learning (ML) applications for mental health within Human-Computer Interaction (HCI) literature | Privacy concerns with data collection and use Algorithmic bias due to lack of representative datasets Ethical responsibility in AI decision-making Digital exclusion risks for vulnerable populations | Autonomy/informed consent  Justice/equity  Privacy/confidentiality  Transparency/explainability  Scientific integrity/validity | Focus on real-world applications of ML for mental health diagnosis, monitoring, and prediction Highlights human-centered, multidisciplinary approaches for system development |
| (Torous et al. 2021) | Comprehensive review of digital psychiatry technologies including apps, social media, chatbots, and virtual reality | Privacy and data security concerns Bias in AI systems  Transparency in algorithms Clinical responsibility Risk of over-reliance on technology | Beneficence/non-maleficence  Autonomy/informed consent  Justice/equity  Privacy/confidentiality  Transparency/explainability  Scientific integrity/validity | Integration into clinical workflow Healthcare systems adoption Need for standardized protocols Training needs for clinicians Need for regulatory oversight |
| (Upreti et al. 2024) | Review of trustworthy machine learning in the context of security, privacy, and federated learning | Security vulnerabilities in ML models Privacy issues in AI systems Bias in ML algorithms Lack of robustness | Autonomy/informed consent  Justice/equity  Privacy/confidentiality  Transparency/explainability  Scientific integrity/validity | Focus on federated learning to enhance privacy and security Need for unified ethical frameworks in AI |
| (Vale 2024) | Investigation into moral entrepreneurship and the ethics of AI in digital psychiatry | Bias in AI systems Lack of transparency in AI/ML algorithms Exclusion of non-experts from ethical decision-making | Autonomy/informed consent  Justice/equity  Privacy/confidentiality  Transparency/explainability  Scientific integrity/validity | Regulation gaps in digital psychiatry Professionalization of ethical standards Role of researchers in shaping AI ethics |
| (Wang 2022) | Ethical analysis of epistemic injustice in computational psychiatry, with a focus on addiction research | Testimonial injustice through silencing patient perspectives in data-driven models Hermeneutic injustice caused by lack of shared interpretive tools in theory-driven models Systemic privileging of computational methods over subjective experiences | Beneficence/non-maleficence  Autonomy/informed consent  Justice/equity  Transparency/explainability  Scientific integrity/validity | Application of data-driven and theory-driven computational models to addiction research Emphasis on integrating patient perspectives into psychiatric decision-making |
| (Wiese and Friston 2022) | Theoretical analysis examining AI ethics in computational psychiatry and ethics of consciousness | Privacy and data security concerns  Risk of biological reductionism ignoring psychosocial factors,  Risk of ignoring conscious experience  Algorithmic bias and fairness issues Concerns about transparency and explainability  Risk of replacing human judgment | Beneficence/non-maleficence  Autonomy/informed consent  Justice/equity  Privacy/confidentiality  Transparency/explainability  Scientific integrity/validity | Focus on theoretical frameworks and considerations rather than specific implementation settings |
| (Wouters et al. 2024) | Ethical analysis of incorporating polygenic scores into clinical psychiatric practice | Risk of stigma and discrimination Misinterpretation of genetic information Lack of informed consent processes Equity concerns due to data biases Psychological harm from genetic risk communication | Beneficence/non-maleficence  Autonomy/informed consent  Justice/equity  Privacy/confidentiality  Scientific integrity/validity | Use of genetic counseling to communicate polygenic risk scores responsibly Focus on patient empowerment through improved understanding of heritability in psychiatric conditions |
| (Wray et al. 2021) | Conceptual review on the utility and limitations of polygenic risk scores (PRS) in clinical psychiatry | Ethical implications of genetic risk communication to patients Privacy and security risks associated with genetic data storage Potential for health disparities in diverse populations Challenges in interpretation of PRS by clinicians and patients Commercial use of PRS without regulation | Autonomy/informed consent  Justice/equity  Privacy/confidentiality  Transparency/explainability  Scientific integrity/validity | Integration of PRS into clinical psychiatry for risk stratification, early intervention, and treatment decisions Applications include psychiatric disorders like schizophrenia and bipolar disorder |
| (Zhang et al. 2023) | Qualitative descriptive study examining mental health professionals' perspectives on AI adoption | Fear of losing human connection in care, Resistance to technology adoption  Limited understanding of AI capabilities  Funding constraints limiting adoption  Concerns about bias in datasets Privacy and security of sensitive mental health data  Challenge of standardization across settings | Beneficence/non-maleficence  Autonomy/informed consent  Justice/equity  Privacy/confidentiality  Transparency/explainability  Scientific integrity/validity | Healthcare organizations across Ontario  Canada including urban and rural settings  Clinical and research contexts, Integration into existing mental health services workflow Educational and training contexts for mental health professionals |
| (Zidaru, Morrow, and Stockley 2021) | Systematic scoping review on PPI in AI-assisted mental health care | Risk of excluding marginalized and underserved populations Ethical challenges of public engagement in AI design Lack of transparency in data use and AI decision-making Bias and inequality in AI systems Data privacy and security concerns | Beneficence/non-maleficence  Autonomy/informed consent  Justice/equity  Transparency/explainability  Scientific integrity/validity | Integration of PPI in AI technology design and implementation Need for co-design and participatory methods Public awareness of AI technologies in mental health care Addressing ethical and safety issues in AI adoption |

*ADHD:* Attention Deficit Hyperactivity Disorder; *AI*: Artificial Intelligence; *APA*: American Psychological Association; *ChatGPT*: Generative Pre-trained Transformer chatbot; *DL*: Deep Learning; *DSS*: Decision Support Systems; *EEG*: Electroencephalography; *EHR*: Electronic Health Records; *EMA*: Ecological Momentary Assessment; *ERS*: Environmental Risk Score; *GDPR*: General Data Protection Regulation; *HCI*: Human-Computer Interaction; *ICU*: Intensive Care Unit; *ML*: Machine Learning; *NLP*: Natural Language Processing; *PPI*: Patient and Public Involvement; *PRS*: Polygenic Risk Sfcore; *SITB*: Self-Injurious Thoughts and Behaviors

**References**

Ahmed, Eman, and Kristien Hens. 2022. “Microbiome in Precision Psychiatry: An Overview of the Ethical Challenges Regarding Microbiome Big Data and Microbiome-Based Interventions.” *AJOB Neuroscience* 13(4):270–86. doi: 10.1080/21507740.2021.1958096.

Arbanas, Goran. 2024. “ChatGPT and Other Chatbots in Psychiatry.” *Archives of Psychiatry Research : An International Journal of Psychiatry and Related Sciences* 60.(2.):137–42. doi: 10.20471/june.2024.60.02.07.

Avula, Vijaya Chandra Reddy, and Sridhar Amalakanti. 2024. “Artificial Intelligence in Psychiatry, Present Trends, and Challenges: An Updated Review.” *Archives of Mental Health* 25(1).

Ball, Tali M., Agnieszka Kalinowski, and Leanne M. Williams. 2020. “Ethical Implementation of Precision Psychiatry.” *Personalized Medicine in Psychiatry* 19–20:100046. doi: 10.1016/j.pmip.2019.05.003.

Bentley, Kate H., Joseph S. Maimone, Erin N. Kilbury, Marshall S. Tate, Hannah Wisniewski, M. Taylor Levine, Regina Roberg, John B. Torous, Matthew K. Nock, and Evan M. Kleiman. 2021. “Practices for Monitoring and Responding to Incoming Data on Self-Injurious Thoughts and Behaviors in Intensive Longitudinal Studies: A Systematic Review.” *Clinical Psychology Review* 90:102098. doi: 10.1016/j.cpr.2021.102098.

Bertl, Markus, Peeter Ross, and Dirk Draheim. 2022. “A Survey on AI and Decision Support Systems in Psychiatry – Uncovering a Dilemma.” *Expert Systems with Applications* 202:117464. doi: 10.1016/j.eswa.2022.117464.

Briganti, Giovanni. 2023. “Artificial Intelligence in Psychiatry.” *Psychiatria Danubina* 35(Suppl 2):15–19.

Chekroud, Adam M., Julia Bondar, Jaime Delgadillo, Gavin Doherty, Akash Wasil, Marjolein Fokkema, Zachary Cohen, Danielle Belgrave, Robert DeRubeis, Raquel Iniesta, Dominic Dwyer, and Karmel Choi. 2021. “The Promise of Machine Learning in Predicting Treatment Outcomes in Psychiatry.” *World Psychiatry* 20(2):154–70. doi: 10.1002/wps.20882.

Chen, IM, YY Chen, SC Liao, and YH Lin. 2022. “Development of Digital Biomarkers of Mental Illness via Mobile Apps for Personalized Treatment and Diagnosis.” *JOURNAL OF PERSONALIZED MEDICINE* 12(6). doi: 10.3390/jpm12060936.

Chen, Zhe Sage, Prathamesh (Param) Kulkarni, Isaac R. Galatzer-Levy, Benedetta Bigio, Carla Nasca, and Yu Zhang. 2022. “Modern Views of Machine Learning for Precision Psychiatry.” *Patterns* 3(11). doi: 10.1016/j.patter.2022.100602.

Clarke, Natasha, Peter Foltz, and Peter Garrard. 2020. “How to Do Things with (Thousands of) Words: Computational Approaches to Discourse Analysis in Alzheimer’s Disease.” *Cortex* 129:446–63. doi: 10.1016/j.cortex.2020.05.001.

D’Alfonso, Simon. 2020. “AI in Mental Health.” *Cyberpsychology* 36:112–17. doi: 10.1016/j.copsyc.2020.04.005.

Davidson, Brittany I. 2022. “The Crossroads of Digital Phenotyping.” *General Hospital Psychiatry* 74:126–32. doi: 10.1016/j.genhosppsych.2020.11.009.

Diaz-Asper, Catherine, Mathias K. Hauglid, Chelsea Chandler, Alex S. Cohen, Peter W. Foltz, and Brita Elvevåg. 2024. “A Framework for Language Technologies in Behavioral Research and Clinical Applications: Ethical Challenges, Implications, and Solutions.” *The American Psychologist* 79(1):79–91. doi: 10.1037/amp0001195.

Dikaios, Katerina, Sheri Rempel, Sri Harsha Dumpala, Sageev Oore, Michael Kiefte, and Rudolf Uher. 2023. “Applications of Speech Analysis in Psychiatry.” *Harvard Review of Psychiatry* 31(1).

D’Souza, RF, M. Mathew, S. Amanullah, JE Thornton, V. Mishra, E. Mohandas, PL Palatty, and KM Surapaneni. 2024. “Navigating Merits and Limits on the Current Perspectives and Ethical Challenges in the Utilization of Artificial Intelligence in Psychiatry - An Exploratory Mixed Methods Study.” *ASIAN JOURNAL OF PSYCHIATRY* 97. doi: 10.1016/j.ajp.2024.104067.

Dube, Anish R., Adrian Jacques H. Ambrose, German Velez, and Mandar Jadhav. n.d. “Real Concerns, Artificial Intelligence: Reality Testing for Psychiatrists.” *International Review of Psychiatry* 1–6. doi: 10.1080/09540261.2024.2363374.

Dwyer, Dominic, and Nikolaos Koutsouleris. 2022. “Annual Research Review: Translational Machine Learning for Child and Adolescent Psychiatry.” *Journal of Child Psychology and Psychiatry* 63(4):421–43. doi: 10.1111/jcpp.13545.

Espejo, Gemma, Wade Reiner, and Michael Wenzinger. 2023. “Exploring the Role of Artificial Intelligence in Mental Healthcare: Progress, Pitfalls, and Promises.” *Cureus* 15(9):e44748. doi: 10.7759/cureus.44748.

Faissner, Mirjam, Eva Kuhn, Regina Müller, and Sebastian Laacke. 2024. “Detecting Your Depression with Your Smartphone? – An Ethical Analysis of Epistemic Injustice in Passive Self-Tracking Apps.” *Ethics and Information Technology* 26(2):28. doi: 10.1007/s10676-024-09765-7.

Fardouly, Jasmine, Ross D. Crosby, and Suku Sukunesan. 2022. “Potential Benefits and Limitations of Machine Learning in the Field of Eating Disorders: Current Research and Future Directions.” *Journal of Eating Disorders* 10(1):66. doi: 10.1186/s40337-022-00581-2.

Farmer, Ryan L., Adam B. Lockwood, Anisa Goforth, and Christopher Thomas. 2024. “Artificial Intelligence in Practice: Opportunities, Challenges, and Ethical Considerations.” *Professional Psychology: Research and Practice* No Pagination Specified-No Pagination Specified. doi: 10.1037/pro0000595.

Fisher, CE. 2024. “The Real Ethical Issues with AI for Clinical Psychiatry.” *INTERNATIONAL REVIEW OF PSYCHIATRY*. doi: 10.1080/09540261.2024.2376575.

Fusar-Poli, Paolo, Mirko Manchia, Nikolaos Koutsouleris, David Leslie, Christiane Woopen, Monica E. Calkins, Michael Dunn, Christophe Le Tourneau, Miia Mannikko, Tineke Mollema, Dominic Oliver, Marcella Rietschel, Eva Z. Reininghaus, Alessio Squassina, Lucia Valmaggia, Lars Vedel Kessing, Eduard Vieta, Christoph U. Correll, Celso Arango, and Ole A. Andreassen. 2022. “Ethical Considerations for Precision Psychiatry: A Roadmap for Research and Clinical Practice.” *European Neuropsychopharmacology* 63:17–34. doi: 10.1016/j.euroneuro.2022.08.001.

Galderisi, Silvana, Paul S. Appelbaum, Neeraj Gill, Piers Gooding, Helen Herrman, Antonio Melillo, Keris Myrick, Soumitra Pathare, Martha Savage, George Szmukler, and John Torous. 2024. “Ethical Challenges in Contemporary Psychiatry: An Overview and an Appraisal of Possible Strategies and Research Needs.” *World Psychiatry* 23(3):364–86. doi: 10.1002/wps.21230.

Gillett, George, and Kate E. A. Saunders. 2019. “Remote Monitoring for Understanding Mechanisms and Prediction in Psychiatry.” *Current Behavioral Neuroscience Reports* 6(2):51–56. doi: 10.1007/s40473-019-00176-3.

Gooding, Piers, and Timothy Kariotis. 2021. “Ethics and Law in Research on Algorithmic and Data-Driven Technology in Mental Health Care: Scoping Review.” *JMIR Ment Health* 8(6):e24668. doi: 10.2196/24668.

Graham, Sarah, Colin Depp, Ellen E. Lee, Camille Nebeker, Xin Tu, Ho-Cheol Kim, and Dilip V. Jeste. 2019. “Artificial Intelligence for Mental Health and Mental Illnesses: An Overview.” *Current Psychiatry Reports* 21(11):116. doi: 10.1007/s11920-019-1094-0.

Gültekin, Mücahit, and Meryem Şahin. 2024. “The Use of Artificial Intelligence in Mental Health Services in Turkey: What Do Mental Health Professionals Think?” *Cyberpsychology: Journal of Psychosocial Research on Cyberspace* 18(1). doi: 10.5817/CP2024-1-6.

Hagendorff, Thilo. 2021. “Linking Human And Machine Behavior: A New Approach to Evaluate Training Data Quality for Beneficial Machine Learning.” *Minds and Machines* 31(4):563–93. doi: 10.1007/s11023-021-09573-8.

Hart, Alexander, Dorota Reis, Elisabeth Prestele, and Nicholas C. Jacobson. 2022. “Using Smartphone Sensor Paradata and Personalized Machine Learning Models to Infer Participants’ Well-Being: Ecological Momentary Assessment.” *J Med Internet Res* 24(4):e34015. doi: 10.2196/34015.

Heinrichs, Bert, and Simon B. Eickhoff. 2020. “Your Evidence? Machine Learning Algorithms for Medical Diagnosis and Prediction.” *Human Brain Mapping* 41(6):1435–44. doi: 10.1002/hbm.24886.

Hurley, Meghan E., Anika Sonig, John Herrington, Eric A. Storch, Gabriel Lázaro-Muñoz, Jennifer Blumenthal-Barby, and Kristin Kostick-Quenet. 2024. “Ethical Considerations for Integrating Multimodal Computer Perception and Neurotechnology.” *Frontiers in Human Neuroscience* 18. doi: 10.3389/fnhum.2024.1332451.

Islam, Md. Monirul, Shahriar Hassan, Sharmin Akter, Ferdaus Anam Jibon, and Md. Sahidullah. 2024. “A Comprehensive Review of Predictive Analytics Models for Mental Illness Using Machine Learning Algorithms.” *Healthcare Analytics* 6:100350. doi: 10.1016/j.health.2024.100350.

Jacobson, Nicholas C., Kate H. Bentley, Ashley Walton, Shirley B. Wang, Rebecca G. Fortgang, Alexander J. Millner, Garth Coombs, Alexandra M. Rodman, and Daniel D. L. Coppersmith. 2020. “Ethical Dilemmas Posed by Mobile Health and Machine Learning in Psychiatry Research.” *Bulletin of the World Health Organization* 98(4):270–76. doi: 10.2471/BLT.19.237107.

Jin, Kevin W., Qiwei Li, Yang Xie, and Guanghua Xiao. 2023. “Artificial Intelligence in Mental Healthcare: An Overview and Future Perspectives.” *British Journal of Radiology* 96(1150):20230213. doi: 10.1259/bjr.20230213.

Kappen, Mitchel, Marie-Anne Vanderhasselt, and George M. Slavich. 2023. “Speech as a Promising Biosignal in Precision Psychiatry.” *Neuroscience & Biobehavioral Reviews* 148:105121. doi: 10.1016/j.neubiorev.2023.105121.

Kirtley, Olivia J., Kasper van Mens, Mark Hoogendoorn, Navneet Kapur, and Derek de Beurs. 2022. “Translating Promise into Practice: A Review of Machine Learning in Suicide Research and Prevention.” *The Lancet Psychiatry* 9(3):243–52. doi: 10.1016/S2215-0366(21)00254-6.

Kleine, Anne-Kathrin, Eva Lermer, Julia Cecil, Anna Heinrich, and Susanne Gaube. 2023. “Advancing Mental Health Care with AI-Enabled Precision Psychiatry Tools: A Patent Review.” *Computers in Human Behavior Reports* 12:100322. doi: 10.1016/j.chbr.2023.100322.

Kolding, Sara, Robert M. Lundin, Lasse Hansen, and Søren Dinesen Østergaard. 2024. “Use of Generative Artificial Intelligence (AI) in Psychiatry and Mental Health Care: A Systematic Review.” *Acta Neuropsychiatrica* 1–14. doi: 10.1017/neu.2024.50.

Koutsouleris, Nikolaos, Tobias U. Hauser, Vasilisa Skvortsova, and Munmun De Choudhury. 2022. “From Promise to Practice: Towards the Realisation of AI-Informed Mental Health Care.” *The Lancet Digital Health* 4(11):e829–40. doi: 10.1016/S2589-7500(22)00153-4.

Leung, Ricky. 2023. “Using AI–ML to Augment the Capabilities of Social Media for Telehealth and Remote Patient Monitoring.” *Healthcare* 11(12). doi: 10.3390/healthcare11121704.

Levkovich, Inbar, Shiri Shinan-Altman, and Zohar Elyoseph. 2024. “Can Large Language Models Be Sensitive to Culture Suicide Risk Assessment?”

Lewis, Anna C. F., Rex L. Chisholm, John J. Connolly, Edward D. Esplin, Joe Glessner, Adam Gordon, Robert C. Green, Hakon Hakonarson, Margaret Harr, Ingrid A. Holm, Gail P. Jarvik, Elizabeth Karlson, Eimear E. Kenny, Leah Kottyan, Niall Lennon, Jodell E. Linder, Yuan Luo, Lisa J. Martin, Emma Perez, Megan J. Puckelwartz, Laura J. Rasmussen-Torvik, Maya Sabatello, Richard R. Sharp, Jordan W. Smoller, Rene Sterling, Shannon Terek, Wei-Qi Wei, and Stephanie M. Fullerton. 2024. “Managing Differential Performance of Polygenic Risk Scores across Groups: Real-World Experience of the eMERGE Network.” *The American Journal of Human Genetics* 111(6):999–1005. doi: 10.1016/j.ajhg.2024.04.005.

Manchia, Mirko, Claudia Pisanu, Alessio Squassina, and Bernardo Carpiniello. 2020. “Challenges and Future Prospects of Precision Medicine in Psychiatry.” *Pharmacogenomics and Personalized Medicine* 13(null):127–40. doi: 10.2147/PGPM.S198225.

Mazor, Matan, Simon Brown, Anna Ciaunica, Athena Demertzi, Johannes Fahrenfort, Nathan Faivre, Jolien C. Francken, Dominique Lamy, Bigna Lenggenhager, Michael Moutoussis, Marie-Christine Nizzi, Roy Salomon, David Soto, Timo Stein, and Nitzan Lubianiker. 2023. “The Scientific Study of Consciousness Cannot and Should Not Be Morally Neutral.” *Perspectives on Psychological Science* 18(3):535–43. doi: 10.1177/17456916221110222.

McCradden, Melissa, Katrina Hui, and Daniel Z. Buchman. 2023. “Evidence, Ethics and the Promise of Artificial Intelligence in Psychiatry.” *Journal of Medical Ethics* 49(8):573. doi: 10.1136/jme-2022-108447.

Monaco, Francesco, Annarita Vignapiano, Martina Piacente, Claudio Pagano, Carlo Mancuso, Luca Steardo, Alessandra Marenna, Federica Farina, Gianvito Petrillo, Stefano Leo, Emanuela Ferrara, Stefania Palermo, Vassilis Martiadis, Marco Solmi, Alessio Maria Monteleone, Alessio Fasano, and Giulio Corrivetti. 2024. “An Advanced Artificial Intelligence Platform for a Personalised Treatment of Eating Disorders.” *Frontiers in Psychiatry* 15. doi: 10.3389/fpsyt.2024.1414439.

Monosov, Ilya E., Jan Zimmermann, Michael J. Frank, Mackenzie W. Mathis, and Justin T. Baker. 2024. “Ethological Computational Psychiatry: Challenges and Opportunities.” *Current Opinion in Neurobiology* 86:102881. doi: 10.1016/j.conb.2024.102881.

Monteith, Scott, Tasha Glenn, John R. Geddes, Eric D. Achtyes, Peter C. Whybrow, and Michael Bauer. 2023. “Challenges and Ethical Considerations to Successfully Implement Artificial Intelligence in Clinical Medicine and Neuroscience: A Narrative Review.” *Pharmacopsychiatry* 56(6):209–13. doi: 10.1055/a-2142-9325.

Morley, Jessica, Caio C. V. Machado, Christopher Burr, Josh Cowls, Indra Joshi, Mariarosaria Taddeo, and Luciano Floridi. 2020. “The Ethics of AI in Health Care: A Mapping Review.” *Social Science & Medicine* 260:113172. doi: 10.1016/j.socscimed.2020.113172.

Murray, Graham K., Tian Lin, Jehannine Austin, John J. McGrath, Ian B. Hickie, and Naomi R. Wray. 2021. “Could Polygenic Risk Scores Be Useful in Psychiatry?: A Review.” *JAMA Psychiatry* 78(2):210–19. doi: 10.1001/jamapsychiatry.2020.3042.

Ortiz, Abigail, and Benoit H. Mulsant. 2024. “Beyond Step Count: Are We Ready to Use Digital Phenotyping to Make Actionable Individual Predictions in Psychiatry?” *J Med Internet Res* 26:e59826. doi: 10.2196/59826.

Ostojic, Dijana, Paris Alexandros Lalousis, Gary Donohoe, and Derek W. Morris. 2024. “The Challenges of Using Machine Learning Models in Psychiatric Research and Clinical Practice.” *European Neuropsychopharmacology* 88:53–65. doi: 10.1016/j.euroneuro.2024.08.005.

Oudin, Antoine, Redwan Maatoug, Alexis Bourla, Florian Ferreri, Olivier Bonnot, Bruno Millet, Félix Schoeller, Stéphane Mouchabac, and Vladimir Adrien. 2023. “Digital Phenotyping: Data-Driven Psychiatry to Redefine Mental Health.” *Journal of Medical Internet Research* 25(1):e44502. doi: 10.2196/44502.

Parziale, Andrea, and Deborah Mascalzoni. 2022. “Digital Biomarkers in Psychiatric Research: Data Protection Qualifications in a Complex Ecosystem.” *Frontiers in Psychiatry* 13.

Pavlopoulos, Adrianos, Theodoros Rachiotis, and Ilias Maglogiannis. 2024. “An Overview of Tools and Technologies for Anxiety and Depression Management Using AI.” *Applied Sciences* 14(19). doi: 10.3390/app14199068.

Rahul, Jagdeep, Diksha Sharma, Lakhan Dev Sharma, Umakanta Nanda, and Achintya Kumar Sarkar. 2024. “A Systematic Review of EEG Based Automated Schizophrenia Classification through Machine Learning and Deep Learning.” *Frontiers in Human Neuroscience* 18.

Rocheteau, Emma. 2023. “On the Role of Artificial Intelligence in Psychiatry.” *The British Journal of Psychiatry* 222(2):54–57. doi: 10.1192/bjp.2022.132.

Saheb, Tahereh, Tayebeh Saheb, and David O. Carpenter. 2021. “Mapping Research Strands of Ethics of Artificial Intelligence in Healthcare: A Bibliometric and Content Analysis.” *Computers in Biology and Medicine* 135:104660. doi: 10.1016/j.compbiomed.2021.104660.

Sahin, D., L. Kambeitz-Ilankovic, S. Wood, D. Dwyer, R. Upthegrove, R. Salokangas, S. Borgwardt, P. Brambilla, E. Meisenzahl, S. Ruhrmann, F. Schultze-Lutter, R. Lencer, A. Bertolino, C. Pantelis, N. Koutsouleris, J. Kambeitz, and PRONIA Study Grp. 2024. “Algorithmic Fairness in Precision Psychiatry: Analysis of Prediction Models in Individuals at Clinical High Risk for Psychosis.” *BRITISH JOURNAL OF PSYCHIATRY* 224(2):55–65. doi: 10.1192/bjp.2023.141.

Shen, Francis X., Benjamin C. Silverman, Patrick Monette, Sara Kimble, Scott L. Rauch, and Justin T. Baker. 2022. “An Ethics Checklist for Digital Health Research in Psychiatry: Viewpoint.” *J Med Internet Res* 24(2):e31146. doi: 10.2196/31146.

Singhal, Sorabh, Danielle L. Cooke, Ricardo I. Villareal, Joel J. Stoddard, Chen-Tan Lin, and Allison G. Dempsey. 2024. “Machine Learning for Mental Health: Applications, Challenges, and the Clinician’s Role.” *Current Psychiatry Reports*. doi: 10.1007/s11920-024-01561-w.

Skorburg, Joshua August, Kieran O’Doherty, and Phoebe Friesen. 2024. “Persons or Data Points? Ethics, Artificial Intelligence, and the Participatory Turn in Mental Health Research.” *American Psychologist* 79(1):137–49. doi: 10.1037/amp0001168.

Smith, William R., Paul S. Appelbaum, Matthew S. Lebowitz, Sinan Gülöksüz, Monica E. Calkins, Christian G. Kohler, Raquel E. Gur, and Ran Barzilay. 2023. “The Ethics of Risk Prediction for Psychosis and Suicide Attempt in Youth Mental Health.” *The Journal of Pediatrics* 263:113583. doi: 10.1016/j.jpeds.2023.113583.

Starke, Georg, Eva De Clercq, Stefan Borgwardt, and Bernice Simone Elger. 2021. “Computing Schizophrenia: Ethical Challenges for Machine Learning in Psychiatry.” *Psychological Medicine* 51(15):2515–21. doi: 10.1017/S0033291720001683.

Starke, Georg, Eva De Clercq, and Bernice S. Elger. 2021. “Towards a Pragmatist Dealing with Algorithmic Bias in Medical Machine Learning.” *Medicine, Health Care and Philosophy* 24(3):341–49. doi: 10.1007/s11019-021-10008-5.

Stein, Olivia A., and Audrey Prost. 2024. “Exploring the Societal Implications of Digital Mental Health Technologies: A Critical Review.” *SSM - Mental Health* 6:100373. doi: 10.1016/j.ssmmh.2024.100373.

Straw, Isabel. 2021. “Ethical Implications of Emotion Mining in Medicine.” *Health Policy and Technology* 10(1):191–95. doi: 10.1016/j.hlpt.2020.11.006.

Sultan, Mubashir, Christin Scholz, and Wouter van den Bos. 2023. “Leaving Traces behind: Using Social Media Digital Trace Data to Study Adolescent Wellbeing.” *Computers in Human Behavior Reports* 10:100281. doi: 10.1016/j.chbr.2023.100281.

Tabb, Kathryn, and Maël Lemoine. 2021. “The Prospects of Precision Psychiatry.” *Theoretical Medicine and Bioethics* 42(5):193–210. doi: 10.1007/s11017-022-09558-3.

Tejavibulya, Link, Max Rolison, Siyuan Gao, Qinghao Liang, Hannah Peterson, Javid Dadashkarimi, Michael C. Farruggia, C. Alice Hahn, Stephanie Noble, Sarah D. Lichenstein, Angeliki Pollatou, Alexander J. Dufford, and Dustin Scheinost. 2022. “Predicting the Future of Neuroimaging Predictive Models in Mental Health.” *Molecular Psychiatry* 27(8):3129–37. doi: 10.1038/s41380-022-01635-2.

Terra, Mohamed, Mohamed Baklola, Shaimaa Ali, and Karim El-Bastawisy. 2023. “Opportunities, Applications, Challenges and Ethical Implications of Artificial Intelligence in Psychiatry: A Narrative Review.” *The Egyptian Journal of Neurology, Psychiatry and Neurosurgery* 59(1):80. doi: 10.1186/s41983-023-00681-z.

Thieme, Anja, Danielle Belgrave, and Gavin Doherty. 2020. “Machine Learning in Mental Health: A Systematic Review of the HCI Literature to Support the Development of Effective and Implementable ML Systems.” *ACM Trans. Comput.-Hum. Interact.* 27(5):34:1-34:53. doi: 10.1145/3398069.

Torous, John, Sandra Bucci, Imogen H. Bell, Lars V. Kessing, Maria Faurholt-Jepsen, Pauline Whelan, Andre F. Carvalho, Matcheri Keshavan, Jake Linardon, and Joseph Firth. 2021. “The Growing Field of Digital Psychiatry: Current Evidence and the Future of Apps, Social Media, Chatbots, and Virtual Reality.” *World Psychiatry* 20(3):318–35. doi: 10.1002/wps.20883.

Upreti, Ramesh, Pedro G. Lind, Ahmed Elmokashfi, and Anis Yazidi. 2024. “Trustworthy Machine Learning in the Context of Security and Privacy.” *International Journal of Information Security* 23(3):2287–2314. doi: 10.1007/s10207-024-00813-3.

Vale, Mira D. 2024. “Moral Entrepreneurship and the Ethics of Artificial Intelligence in Digital Psychiatry.” *Socius* 10:23780231241259641. doi: 10.1177/23780231241259641.

Wang, Junyi. 2022. “The Role of the Dominant Attribution Style and Daily Hassles in the Symptoms of Depression and Anxiety.” *Psychology, Health & Medicine* 27(7):1637–48. doi: 10.1080/13548506.2021.2017471.

Wiese, Wanja, and Karl J. Friston. 2022. “AI Ethics in Computational Psychiatry: From the Neuroscience of Consciousness to the Ethics of Consciousness.” *Behavioural Brain Research* 420:113704. doi: 10.1016/j.bbr.2021.113704.

Wouters, Roel H. P., Marte Z. van der Horst, Cora M. Aalfs, Janita Bralten, Jurjen J. Luykx, and Janneke R. Zinkstok. 2024. “The Ethics of Polygenic Scores in Psychiatry: Minefield or Opportunity for Patient-Centered Psychiatry?” *Psychiatric Genetics* 34(2).

Wray, Naomi R., Tian Lin, Jehannine Austin, John J. McGrath, Ian B. Hickie, Graham K. Murray, and Peter M. Visscher. 2021. “From Basic Science to Clinical Application of Polygenic Risk Scores: A Primer.” *JAMA Psychiatry* 78(1):101–9. doi: 10.1001/jamapsychiatry.2020.3049.

Zhang, Melody, Jillian Scandiffio, Sarah Younus, Tharshini Jeyakumar, Inaara Karsan, Rebecca Charow, Mohammad Salhia, and David Wiljer. 2023. “The Adoption of AI in Mental Health Care–Perspectives From Mental Health Professionals: Qualitative Descriptive Study.” *JMIR Form Res* 7:e47847. doi: 10.2196/47847.

Zidaru, Teodor, Elizabeth M. Morrow, and Rich Stockley. 2021. “Ensuring Patient and Public Involvement in the Transition to AI-Assisted Mental Health Care: A Systematic Scoping Review and Agenda for Design Justice.” *Health Expectations* 24(4):1072–1124. doi: 10.1111/hex.13299.
